# Supplementary material for: Molecular and supramolecular adaptation by coupled stimuli
Source: Nat Commun. 2024 Jul 7;15:5695. doi: 10.1038/s41467-024-50029-1 (PMC11228013; doi:10.1038/s41467-024-50029-1)
Supplement: Supplementary file 1 — Supplementary Information [file 41467_2024_50029_MOESM1_ESM.pdf]

Supplementary Information

Molecular and Supramolecular Adaptation by coupled Stimuli

Torsten Dünnebacke,[a] Niklas Niemeyer,[a,b] Sebastian Baumert,[a] Sebastian Hochstädt,[c] Lorenz Borsdorf,[a] Michael Ryan Hansen,[c]* Johannes Neugebauer,[a,b]* Gustavo Fernández[a]*

[a] T. Dünnebacke, N. Niemeyer, S. Baumert, L. Borsdorf, Prof. Dr. J. Neugebauer, Prof. Dr. G. Fernández

Universität Münster, Organisch-Chemisches Institut, Corrensstraße 36, 48149 Münster (Germany).

[b] N. Niemeyer, Prof. Dr. J. Neugebauer

Universität Münster, Center for Multiscale Theory and Computation, Corrensstraße 36, 48149 Münster (Germany).

[c] S. Hochstädt, Prof. Dr. M. R. Hansen

Universität Münster, Institut für Physikalische Chemie, Corrensstraße 28/30, 48149 Münster (Germany).

E-mail: fernandg@uni-muenster.de; j.neugebauer@uni-muenster.de; mhansen@uni-muenster.de

Contents

1 Experimental part 2

1.1 Materials and Methods 2

2 Nucleation-Elongation model for Cooperative Supramolecular Polymerizations 16

3 Supplementary Figures 17

3.1 Solvent-dependent spectroscopic behavior 17

3.2 Stimuli-responsive behavior 18

3.3 Self-Assembly in aliphatic Solvents 30

3.4 Supramolecular adaptive behavior 35

4. Supplementary references 42

1 Experimental part

1.1 Materials and Methods

General Procedures: All solvents were dried according to standard procedures. Reagents were used as purchased. All air-sensitive reactions were carried out under argon or nitrogen atmosphere. Flash chromatography was performed using silica gel (Merck Silica 60, particle size 0.04–0.063 nm).

NMR measurements: ^{1}H- and ^{13}C-NMR spectra were recorded either on a Bruker Avance II 400 (^{1}H: 400 MHz; ^{13}C: 100.6 MHz) or a Bruker NEO 400 (^{1}H: 400 MHz; ^{13}C: 100.6 MHz). Additional 1D-^{1}H-, ^{13}C-, as well as 2D-H,H-COSY , C,H-gHSQC-, C,H-gHMBC-, F,H-HOESY- and H,H-ROESY spectra were recorded on an Agilent DD2 500 (^{1}H: 500 MHz, ^{13}C: 125 MHz) and an Agilent DD2 600 (^{1}H: 600 MHz, ^{13}C: 150 MHz) at a standard temperature of 298 K in deuterated solvents. The recorded spectra were referenced to the remaining resonance signals of the deuterated solvents ($CDCl_3$: 7.26 ppm (^{1}H); DCM: 5.32 ppm (^{1}H); DMSO: 2.50 ppm (^{1}H,); THF: 1.73 ppm (^{1}H); D_2O: 4.79 (^{1}H); MeOD: 4.87 ppm (^{1}H)). The coupling constant J of the measured spin multiplets is given in Hertz (Hz) and the chemical shifts δ are given in reference to the chemical shift of trimethylsilane (0 ppm). The abbreviations used to analyze the recorded spectra are: *s* (singlet), *d* (doublet), *t* (triplet), *q* (quartet), *m* (multiplet).

Solid-state NMR measurements: Solid-state NMR experiments were carried out on a Bruker Avance NEO (ν_L(^{1}H) = 500.39 MHz, 11.74 T) spectrometer with a 4 mm H/F/X MAS DVT probe. Samples were packed into 4 mm ZrO_2 rotors, sealed with a Vespel© top and bottom caps. The 2D ^{13}C{^{1}H} HETCOR NMR experiments were carried out utilizing MAS frequencies of 10.0 kHz and adamantane was used for optimizing pulse lengths for CP conditions and as an external reference (δ(^{1}H) = 1.85 ppm, δ(^{13}C) = 29.47 ppm). To identify ^{13}C showing intramolecular dipolar interactions with ^{1}H, a short CP contact time of τ_{CP} = 0.5 ms was employed. To investigate intermolecular dipolar interactions, spectra with τ_{CP} = 4 ms were obtained. The STATES-TPPI[1] procedure was used to obtain phase sensitive 2D spectra recording 64 rotor synchronized t_1 increments in the indirect dimension. Homonuclear dipolar decoupling was applied with a ^{1}H RF field strength of 83.33 kHz during acquisition. Gaussian line broadening (gb = 0.1, lb = 80 Hz) and a QSINE (SSB = 3) window functions were utilized in the direct and indirect dimension before Fourier transform.

Mass spectroscopy: MALDI-mass spectra were recorded on an Autoflex Speed manufactured by Bruker Daltronics. A SmartBeamTM NdYAF-Laser with a wavelength of 335 nm was used. The signals are described by their mass/charge ratio (*m*/*z*) in Da. ESI accurate masses were measured on a MicroTof (Bruker Daltronics, Bremen) with loop injection. Mass calibration was performed using sodium formate cluster ions immediately filled by the sample in a quasi-internal calibration. ESI mass spectra were recorded on an LTQ Orbitap LTQ XL (Thermo-Fisher Scientific, Bremen) with nano spray (alternatively HPLC, loop injection, syringe pump).

UV-Vis spectroscopy: All UV-Vis spectra were recorded on a V-770, V-750 and a V-730 by the company JASCO or a Cary 4000 by the company Agilent with a spectral bandwidth of 1.0 nm and a scan rate of either 400 or 1000 nm min^{-1}. Glass cuvettes with a path length of 1 cm, 0.5 cm, 1 mm and 0.1 mm were used. All measurements have been conducted in solvents of spectroscopic grade from commercial sources.

Fluorescence spectroscopy: The fluorescence spectra were recorded on a FP-8500 or a FP-8550 by the company JASCO using quartz cuvettes (SUPRASIL®, Hellma) of 1 cm thickness and lamp intensity of the 75 W xenon lamp (type Ushio UXL-75 XE).

FT-IR spectroscopy: All FT-IR spectra were recorded on a JASCO-FT-IR-4600. Solid state FT-IR was measured using a JASCO ATR Pro-One single reflector ATR unit.

Dynamic Light Scattering: All DLS spectra have been recorded on a CGS-3 Compact Goniometer System manufactured by ALV GmbH, equipped with a HeNe Laser with a wavelength of 632.8 nm (22 mW) and an ALV/LSE-5004 Digital Correlator by ALV GmbH.

Atomic force microscopy: The AFM images have been recorded on a Multimode®8 SPM System manufactured by Bruker AXS. The used cantilevers were AC200TS by Oxford Instruments with an average spring constant of 42 N m^{-1}, an average frequency of 150 kHz, an average length of 200 µM, an average width of 40 µM and an average tip radius of 7 nm. All samples were immobilized either on a HOPG or Si-Wafer surface. The samples were applied in solution by placing 10 µL of a 10 µM solution on the substrate for 12 s followed by spin-coating at a spin velocity of 2000 rpm.

Scanning electron microscopy: The SEM images have been recorded on a *Thermo Fisher Scientific Phenom ProX* Desktop SEM by Thermo Fisher Scientific. The individual images have been recorded using a zoom between 22500× and 300× with either a BSD or SED detector and an acceleration voltage of either 5 or 10 kV. A back scattered-electron detector (BSD) or a secondary-electron detector (SED) were used. The corresponding samples were prepared by drop-casting a small volume (10 µL) of the sample in solution on a Si-wafer surface followed by slow solvent evaporation under ambient conditions.

Irradiation Methods: Irradiation-based experiments were performed with LEDs by *Conrad Electronics* as follows: HighPower-LED Grün 87 lm 130° 3.8 V 1000 mA Roschwege LSC-G (λ_{LED} = 520 nm), HighPower-LED Blau 31 lm 130° 2.3 V 700 mA Roschwege LSC-B (λ_{LED} = 465 nm), HighPower-LED 365 nm 100° 4.1 V 700 mA Roschwege (λ_{LED} = 365 nm) and HighPower-LED 405 nm 100° 3.8 V 1000 mA Roschwege (λ_{LED} = 405 nm).

Theoretical calculations: All optimized geometries were obtained with GFN2-xTB 6.4.1.[2] Electronic energies E_{tot} for the protonated monomers were calculated using TURBOMOLE 7.5.1[3] and PW6B95[4] as an approximation for the exchange-correlation functional, a def2-TZVP[5] basis set and D3 dispersion correction[6] with Becke-Johnson damping.[7] Solvation free energies (G_{solv}, 298K, DCM) were obtained with COSMO-RS[8,9] using TURBOMOLE 7.5.1 for the SCF calculation and the BP86[10,11]/def2-TZVP[5] parametrization. Thermostatistical corrections to the free energy (G_{therm}, 298K) were calculated from vibrational frequencies obtained with GFN2-xTB 6.4.1. Final free energies G are obtained as a sum of E_{tot}, G_{solv}, and $G_{therm.}$. Excitation energies and the corresponding orbital analysis have been performed with CAM-B3LYP[12]/def2-TZVP/CPCM[13](CH_2Cl_2) using SERENITY 1.5.2.[14–16]

1.2 Synthesis and characterization

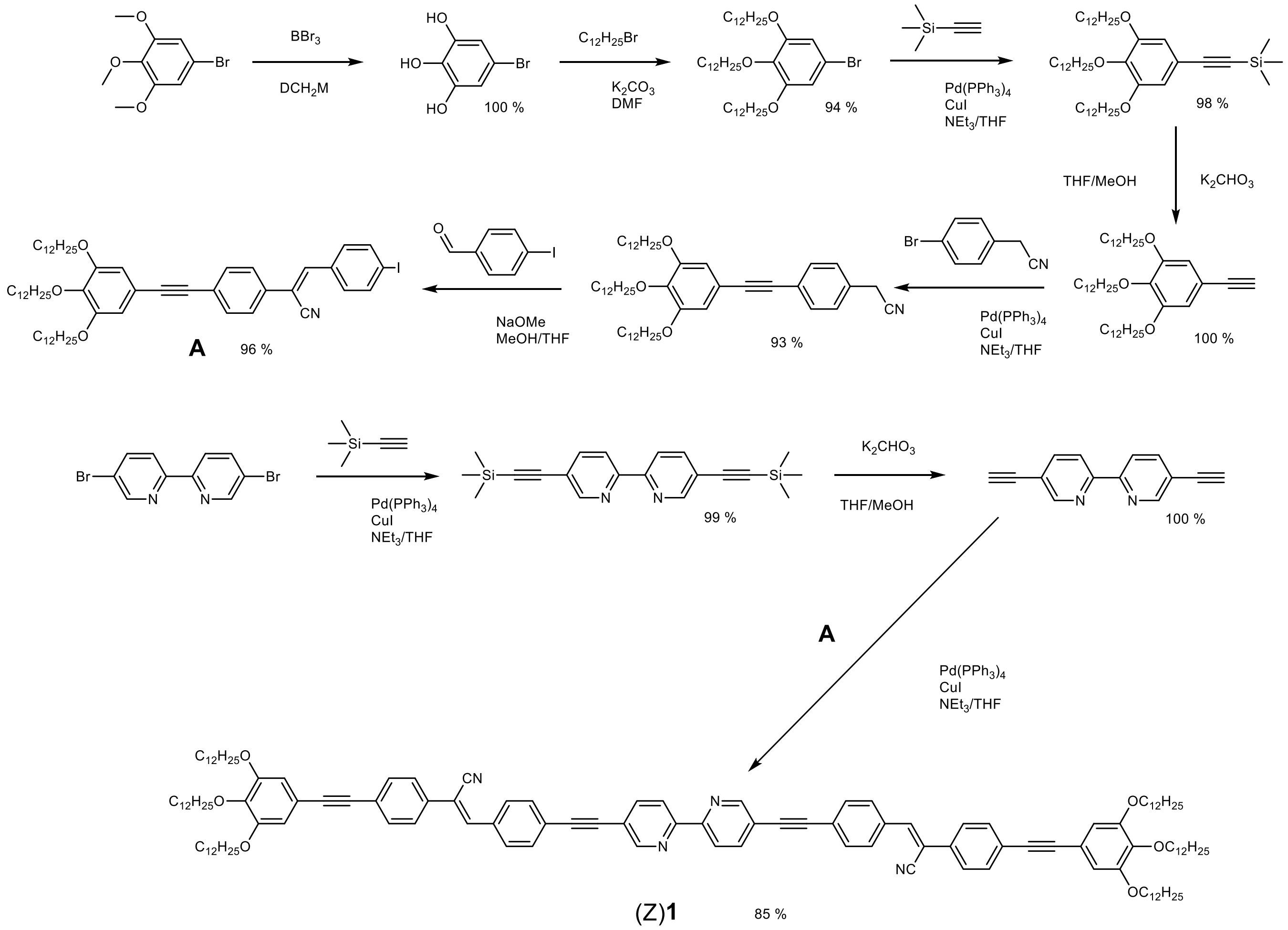

Supplementary Figure 1: Synthetic route towards the target molecule (Z)**1**.

1-Bromo-(3,4,5-trihydroxy)benzol

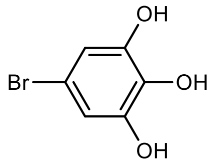

$C_6H_5BrO_3$
205,0070 g/mol

According to a modified literature protocol,[17] 5-Bromo-1,2,3-trimethoxybenzene (6.17 g, 24.95 mmol, 1.00 eq) was dissolved in freshly distilled DCM (120 mL) and degassed with Argon for 15 min. The solution was cooled to -78 °C and stirred for 30 min. At this temperature, BBr_3 (9.47 mL, 99.78 mmol, 4.00 eq) was carefully added dropwise and the reaction mixture was stirred for another 30 min. After slow warming to room temperature the reaction mixture was stirred overnight. A saturated, aqueous solution of NH_4Cl (30 mL) was slowly added to quench the reaction. The phases were separated, the aqueous layer was extracted with EtOAc (3x30 mL) and the combined organic layers were washed with water (3x100 mL). The organic layer was dried over $MgSO_4$ and all volatile compounds were removed under reduced pressure to afford the product as a grey solid (5.09 g, 24.84 mmol, 99 %). This compound was used without further purification. MS (ESI, MeOH): found 202.9355 $[M-H]^-$, calc. for $C_6H_4O_3Br$: 202.9338; **^{1}H-NMR** (DMSO-d_6, 300 MHz, 293 K): δ (ppm) = 9.29 (*s*, br., 2H, -OH), 8.32 (*s*, br., 1H, -OH), 6.40 (*s*, 2H, H_{arom}). The analytical results are in agreement with the data published in the literature.[17]

5-bromo-1,2,3-tris(dodecyloxy)benzene

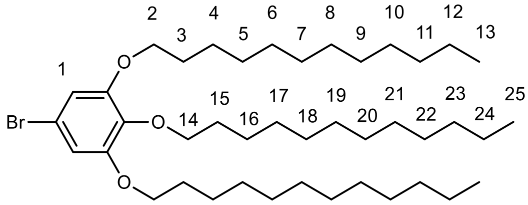

$C_{42}H_{77}BrO_3$
709.9790 g/mol

Following a reaction procedure reported in the literature,[18] 5-Bromo-1,2,3-trihydroxybenzene (2.00 g, 9.76 mmol, 1.00 eq) and 1-Bromododecane (9.37 mL, 9.73 g, 39.02 mmol, 4.00 eq) were dissolved in DMF (50 mL, degassed/dry). K_2CO_3 (8.09 g, 58.54 mmol, 6.00 eq) was added, the reaction mixture was heated up to 110 °C and stirred for 20.5 h. After cooling down to r.t., the mixture was poured on ice water (70 mL) and subsequently extracted with DCM (3×70 mL). The combined organic layers were dried over $MgSO_4$ and all volatile compounds were removed under reduced pressure. The residue was purified by column chromatography (SiO_2, 0-1.5 % EtOAc/Pentane). The desired product was obtained as a colorless wax-like solid (6.53 g, 9.20 mmol, 94 %). **^{1}H-NMR** ($CDCl_3$, 400 MHz, 300 K): δ (ppm) = 6.67 (*s*, 2H, H_1); 3.96 - 3.88 (*m*, 6H, H_2+H_{14}); 1.82 – 1.75 (*m*, 4H, H_3); 1.75 – 1.68 (*m*, 2H, H_{15}); 1.50 – 1.41 (*m*, 6H, H_4+H_{16}); 1.37 – 1.22 (*m*, 48H, H_{5-12}+H_{17-24}); 0.88 (*t*, J = 6.8 Hz, 9H, H_{13}+H_{25}). The analytical results are in good agreement with the data published in the literature.[18]

Trimethyl((3,4,5-tris(dodecyloxy)phenyl)ethynyl)silane

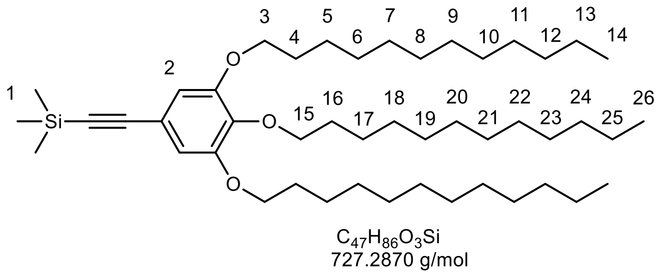

According to a literature procedure,[19] 5-bromo-1,2,3-tris(dodecyloxy)benzene (2.00 g, 2.82 mmol, 1.00 eq.), $Pd(PPh_3)_4$ (162 mg, 5 mol%) and CuI (54 mg, 10 mol%) were dispersed in NEt_3 (18 mL, distilled/degassed) and the mixture was stirred at r.t. for 20 min. Trimethylsilylacetylene (601 µL, 415 mg, 4.23 mmol, 1.5 eq) was slowly added dropwise, the reaction mixture was heated up to 80 °C and stirred for 21 h. After cooling to r.t., the mixture was diluted with DCM and filtered over Celite.® All volatile compounds were removed in vacuo and the residue was purified by column chromatography (SiO_2, 5 % Et_2O/pentane). The product was obtained as a colorless, wax-like solid (2.01 g, 2.76 mmol, 98 %). **^{1}H-NMR** ($CDCl_3$, 400 MHz, 295 K): δ(ppm) = 6.66 (*s*, 2H, H_2); 3.94 (*t*, *J* = 6.5 Hz, 6H, H_3+H_{15}); 1.79 (*p*, *J* = 6.6 Hz, 4H, H_4); 1.75 – 1.67 (*m*, 2H, H_{16}); 1.49 – 1.41 (*m*, 6H, H_5+H_{17}); 1.37 – 1.21 (*m*, 48H, $H_{6\text{-}13}$+$H_{18\text{-}25}$); 0.88 (*t*, *J* = 6.7 Hz, 9H, H_{14}+H_{26}), 0.24 (*s*, 9H, H_1). The analytical results are in good agreement with the data published in the literature.[19]

1,2,3-tris(dodecyloxy)-5-ethynylbenzene

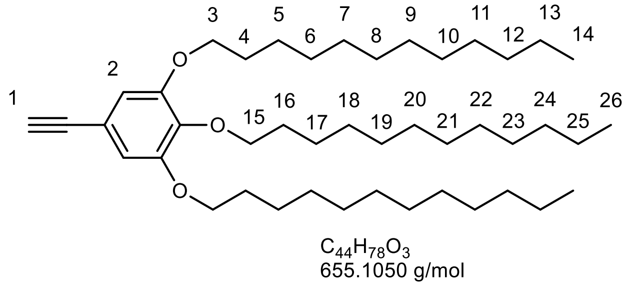

Following a procedure published in the literature,[19] Trimethyl((3,4,5-tris(dodecyloxy)phenyl) ethynyl)silane (1.94 g, 2.67 mmol, 1.00 eq) was dissolved in a 1:1 mixture of THF and MeOH (16 mL). K_2CO_3 (1.29 g, 9.35 mmol, 3.5 eq) was added and the dispersion was stirred at r.t. for 1.5 h. The reaction mixture was poured on water (15 mL) and subsequently extracted with DCM (15 mL, 2×10 mL). The combined organic layers were dried over $MgSO_4$ and the solvent was removed under reduced pressure. The product was obtained as a colorless, wax-like solid and used without further purification (1.75 g, 2.67 mmol, 100 %). **^{1}H-NMR** ($CDCl_3$, 400 MHz, 300 K): δ(ppm) = 6.69 (*s*, 2H, H_2); 3.99 – 3.91 (*m*, 6H, H_3+H_{15}); 2.99 (*s*, 1H, H_1); 1.83 – 1.75 (*m*, 4H, H_4); 1.75 – 1.68 (*m*, 2H, H_{16}); 1.50 – 1.41 (*m*, 6H, H_5+H_{17}); 1.37 – 1.22 (*m*, 48H, $H_{6\text{-}13}$+$H_{18\text{-}25}$); 0.88 (*t*, *J* = 6.8 Hz, 9H, H_{14}+H_{26}). The analytical results are in good agreement with the data published in the literature.[19]

2-(4-((3,4,5-tris(dodecyloxy)phenyl)ethynyl)phenyl)acetonitrile

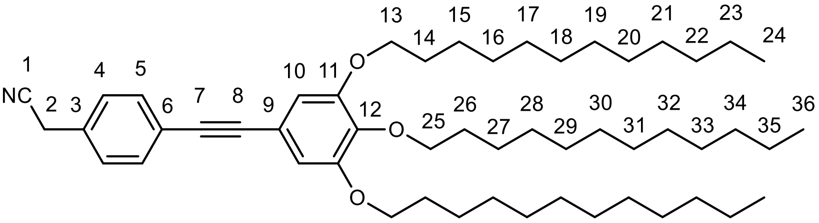

$C_{52}H_{83}NO_3$
770.2400 g/mol

2-(4-Iodophenyl)acetonitrile (617 mg, 2.54 mmol, 1.00 eq), $Pd(PPh_3)_4$ (147 mg, 5 mol%) and CuI (48 mg, 10 mol%) were dispersed in NEt_3 (8 mL, distilled/degassed) and THF (5 mL, distilled/degassed) and subsequently stirred at r.t. for 20 min. 1,2,3-tris(dodecyloxy)-5-ethynylbenzene (1.75 g, 2.67 mmol, 1.05 eq) in THF (5 mL, distilled/degassed) was slowly added dropwise, the reaction mixture was heated up to 50 °C and subsequently stirred for 17.5 h. The reaction mixture was allowed to cool to r.t., diluted with DCM and filtered over Celite.® All volatile compounds were removed under reduced pressure and the residue was purified by column chromatography (SiO_2, 15-20 % Et_2O/pentane). The desired product was obtained as a slightly yellow, wax-like solid (1.82 g, 2.36 mmol, 93 %). %). **MS** (ESI Nanospray, $CHCl_3/CH_3CN$): *m/z* = 792.62675 $[M+Na]^+$, 1563.26834 $[2M+Na]^+$, calculated for: $C_{52}H_{83}NO_3Na^+$ 792.62652, $C_{104}H_{166}N_2O_6Na^+$ 1563.26718. **^{1}H-NMR** ($CDCl_3$, 600 MHz, 299 K): δ(ppm) = 7.52 (*d*, J = 8.4 Hz, 2H, H_5), 7.31 (*d*, J = 8.6 Hz, 2H, H_4), 6.73 (*s*, 2H, H_{10}), 4.00 – 3.96 (*m*, 6H, H_{13}+H_{25}), 3.77 (*s*, 2H, H_2), 1.84 – 1.78 (*m*, 4H, H_{14}), 1.77 – 1.72 (*m*, 2H, H_{26}), 1.51 – 1.44 (*m*, 6H, H_{15}+H_{27}), 1.39 – 1.33 (*m*, 6H, H_{16}+H_{28}), 1.33 – 1.23 (*m*, 42H, H_{17-23}+H_{29-35}), 0.88 (*t*, J = 7.1 Hz, 9H, H_{24}+H_{36}); **^{13}C-NMR** ($CDCl_3$, 151 MHz, 299 K): δ(ppm) = 153.2 (C_{11}), 139.5 (C_{12}), 132.3 (C_5), 129.8 (C_3) , 128.1 (C_4), 123.6 (C_6), 117.6 (C_1), 117.4 (C_9), 110.4 (C_{10}), 90.8 (C_8), 87.3 (C_7), 73.7 (C_{25}), 69.3 (C_{13}), 32.1 (-CH_2-), 32.1 (-CH_2-), 30.5 (C_{26}), 29.9 (-CH_2-), 29.9 (-CH_2-), 29.9 (-CH_2-), 29.9 (-CH_2-), 29.8 (-CH_2-), 29.8 (-CH_2-), 29.7 (-CH_2-), 29.5 (-CH_2-), 29.5 (-CH_2-), 29.5 (-CH_2-), 26.3 (C_{27}), 26.2 (C_{15}), 23.7 (C_2), 22.85, 22.84, 14.26 (C_{24}+C_3).

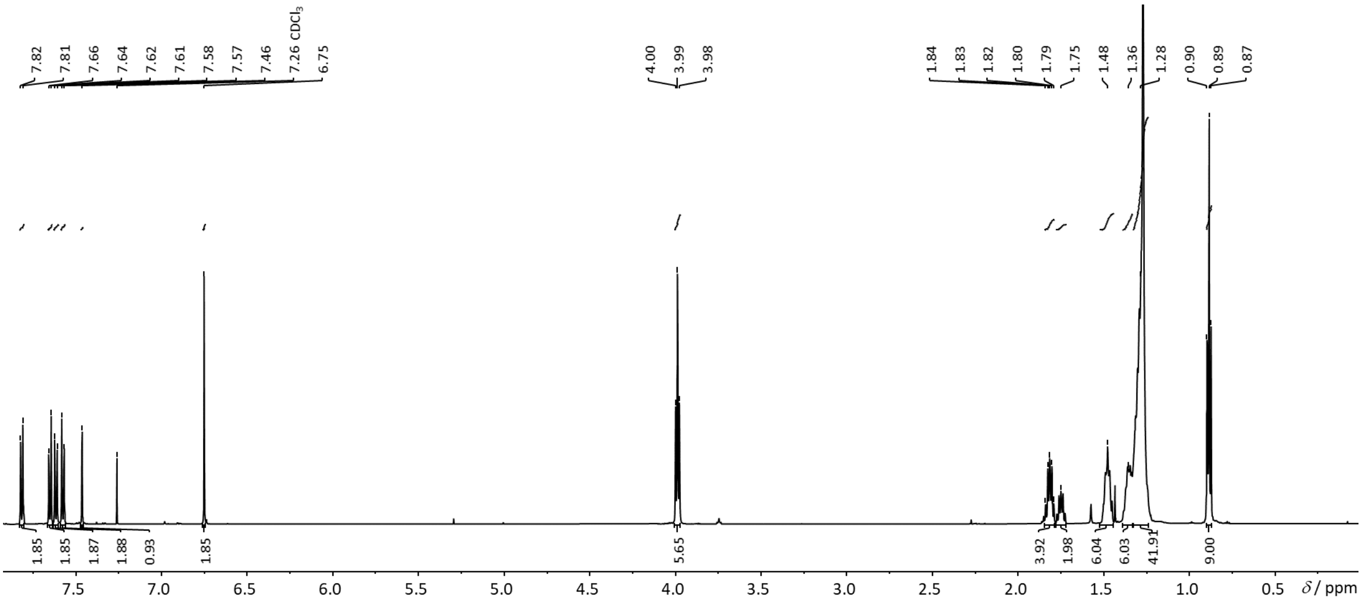

Supplementary Figure 2: ^{1}H-NMR ($CDCl_3$, 600 MHz, 299 K) of 2-(4-((3,4,5-tris(dodecyloxy)phenyl)ethynyl)phenyl)acetonitrile.

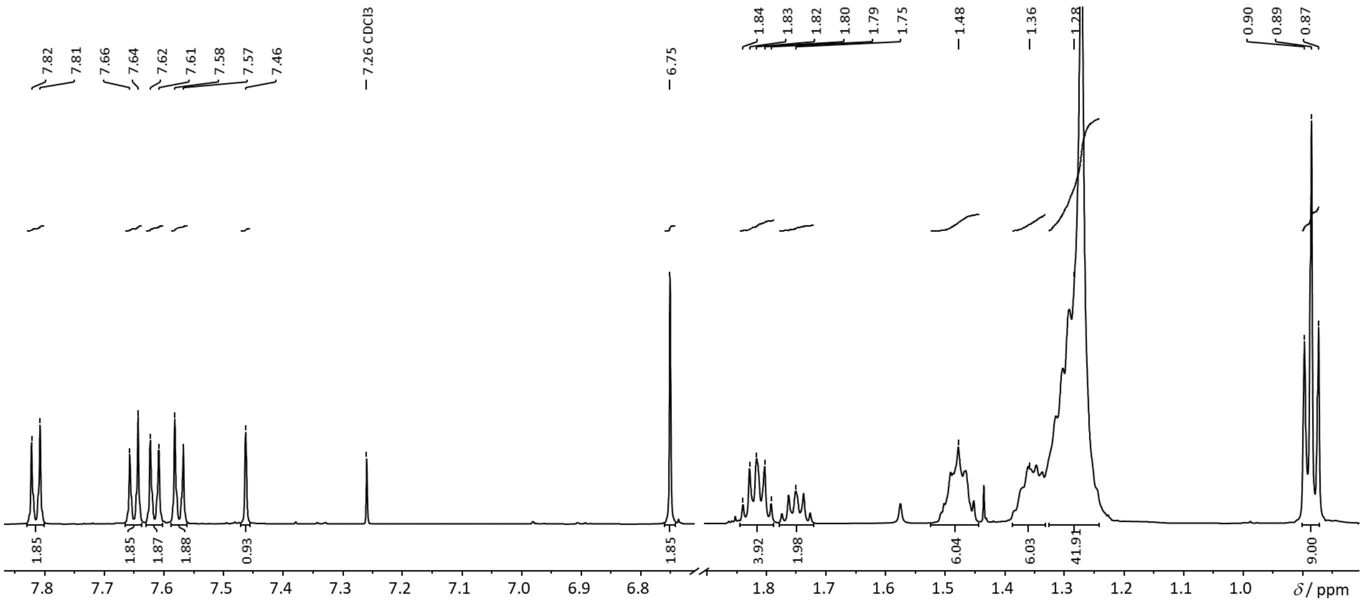

Supplementary Figure 3: Magnification of the aromatic region of the ^{1}H-NMR ($CDCl_3$, 600 MHz, 299 K) of 2-(4-((3,4,5-tris(dodecyloxy) phenyl)ethynyl)phenyl)acetonitrile.

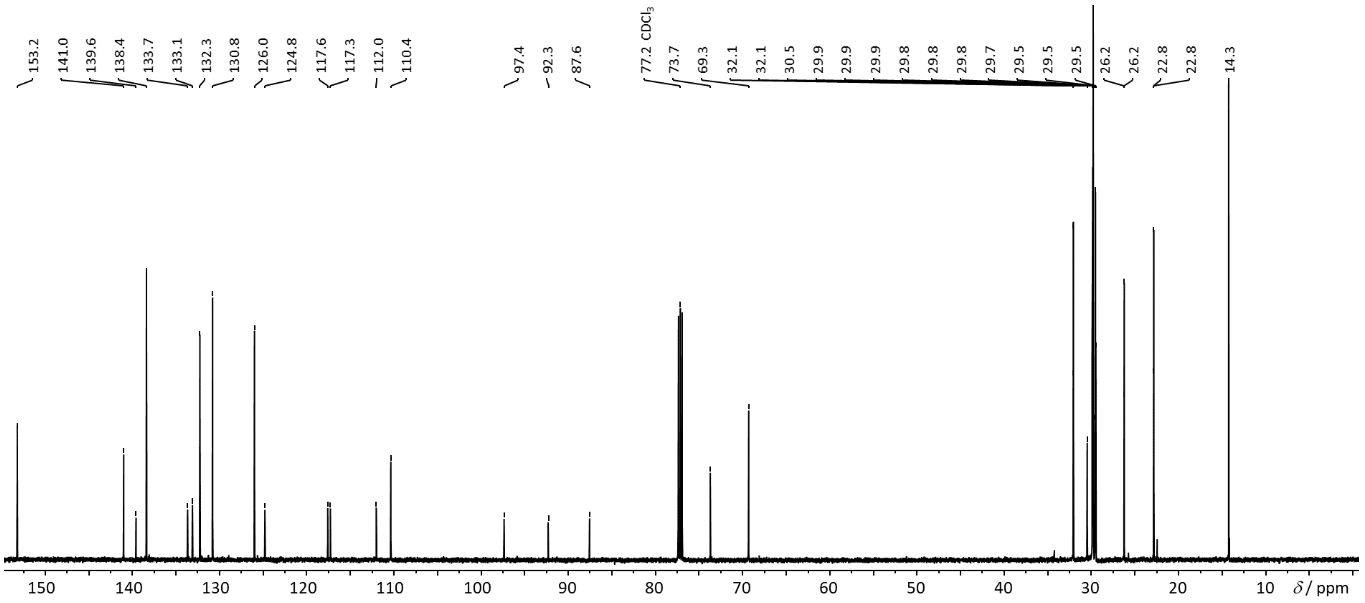

Supplementary Figure 4: ^{13}C-NMR ($CDCl_3$, 151 MHz, 299 K) of 2-(4-((3,4,5-tris(dodecyloxy)phenyl)ethynyl)phenyl)acetonitrile.

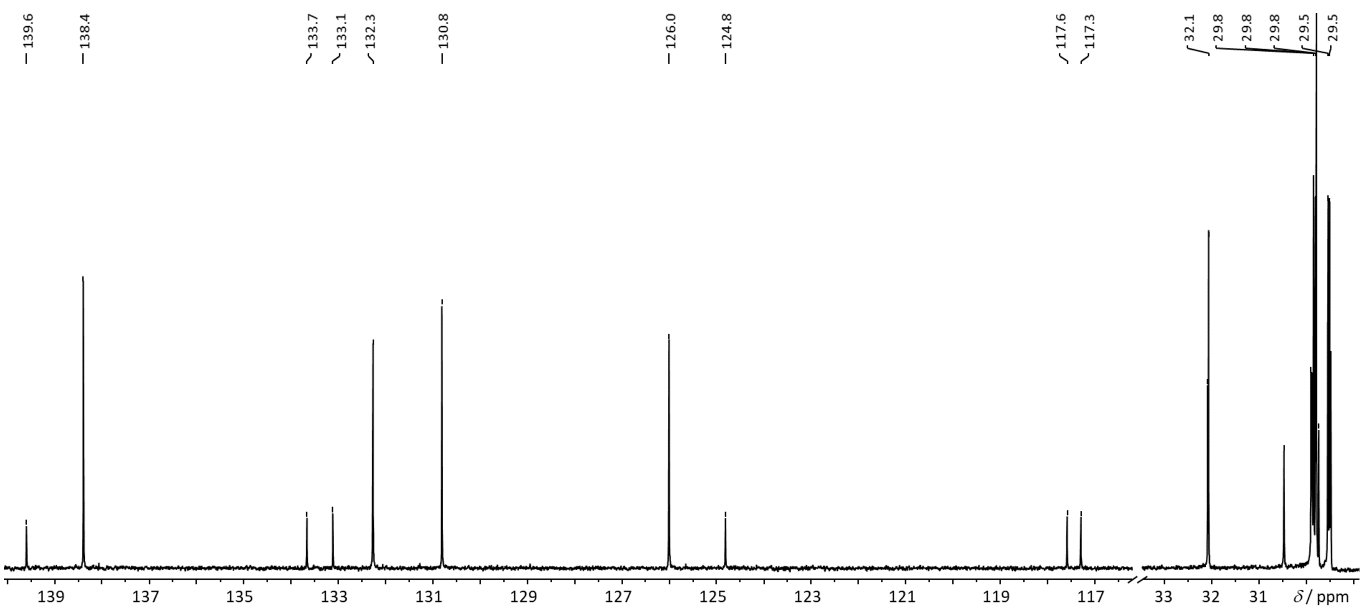

Supplementary Figure 5: Magnification of the aromatic region of the ^{13}C-NMR ($CDCl_3$, 151 MHz, 299 K) of 2-(4-((3,4,5-tris(dodecyloxy) phenyl) ethynyl)phenyl)acetonitrile.

(*Z*)-3-(4-iodophenyl)-2-(4-((3,4,5-tris(dodecyloxy)phenyl)ethynyl)phenyl)acrylonitrile

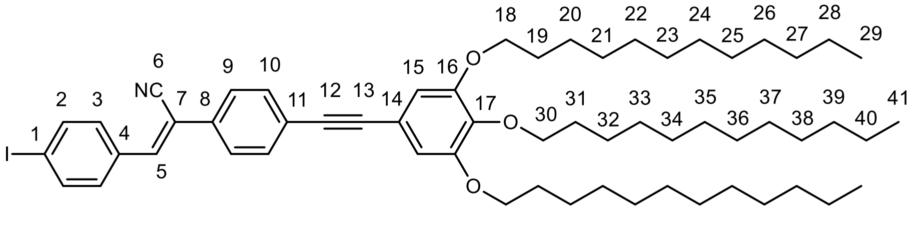

2-(4-((3,4,5-tris(dodecyloxy)phenyl)ethynyl)phenyl)acetonitrile (1.82 g, 2.26 mmol, 1.00 eq) and 4-Iodobenzaldehyde (548 mg, 2.36 mmol, 1.00 eq) were dissolved in MeOH (14 mL) and THF (14 mL). NaOMe (159 mg, 2.83 mmol, 1.20 eq) was next added, the reaction mixture was heated up to 60 °C and stirred for 15 h. After cooling to r.t., the reaction was quenched by slow addition of water (40 mL). It was subsequently extracted with DCM (3×40 mL) and the combined organic layers were dried over $MgSO_4$. All volatile compounds were removed in vacuo and the residue was purified by column chromatography (SiO_2, 7 % Et_2O/pentane). The product was obtained as a yellow solid (2.23 g, 2.6 mmol, 96 %). **MS** (ESI Nanospray, MeOH/$CHCl_3$): *m/z* = 1006.55505 $[M+Na]^+$, 1991.12305 $[2M+Na]^+$, calculated for: $C_{59}H_{86}NO_3INa^+$ 1006.55446, $C_{118}H_{172}N_2O_6Na^+$ 1991.12306. **^{1}H-NMR** ($CDCl_3$, 600 MHz, 299 K): δ(ppm) = 7.82 (*d*, J = 8.5 Hz, 2H, H_2), 7.65 (*d*, J = 8.5 Hz, 2H, H_9), 7.62 (*d*, J = 8.5 Hz, 2H, H_3), 7.57 (*d*, J = 8.5 Hz, 2H, H_{10}), 7.46 (*s*, 1H, H_5), 6.75 (*s*, 2H, H_{15}), 3.99 (*t*, J = 6.5 Hz, 6H, H_{18}+H_{30}), 1.82 (*p*, J = 6.7 Hz, 4H, H_{19}), 1.78 – 1.72 (*m*, 2H, H_{31}), 1.51 – 1.45 (*m*, 6H, H_{20}+H_{32}), 1.39 – 1.33 (*m*, 6H, H_{21}+H_{33}), 1.33 – 1.23 (*m*, 42H, $H_{22\text{-}28}$+$H_{34\text{-}40}$), 0.89 (*t*, J = 7.1 Hz, 9H, H_{29}+H_{41}). **^{13}C-NMR** ($CDCl_3$, 151 MHz, 299 K): δ(ppm) = 153.2 (C_{16}), 141.0 (C_5), 139.6 (C_{17}), 138.4 (C_2), 133.7 (C_8), 133.1 (C_4), 132.3 (C_{10}), 130.8 (C_3), 126.0 (C_9), 124.8 (C_{11}), 117.6 (C_6), 117.3 (C_{14}), 112.0 (C_7), 110.4 (C_{15}), 97.4 (C_1), 92.3 (C_{13}), 87.6 (C_{12}), 73.7 (C_{30}), 69.3 (C_{18}), 32.1 (C_{39}), 32.1 (C_{27}), 30.5 (C_{31}), 29.9 (-CH_2-), 29.9 (-CH_2-), 29.9 (-CH_2-), 29.9 (-CH_2-), 29.8 (-CH_2-), 29.8 (-CH_2-), 29.7 (-CH_2-), 29.5 (-CH_2-), 29.5 (-CH_2-), 29.5 (-CH_2-), 26.3 (C_{32}), 26.2 (C_{20}), 22.8 (C_{40}), 22.8 (C_{28}), 14.30 (C_{29}+C_{41}). To characterize the configuration of the central C=C-bond, the C_{12}-H_{10} coupling constant was determined. The coupling

constant was found to be $^3J_{CN-H}$ = 14.4 Hz, which is in good agreement with the corresponding value for *Z*-configured CN-substituted stilbenes reported in the literature.

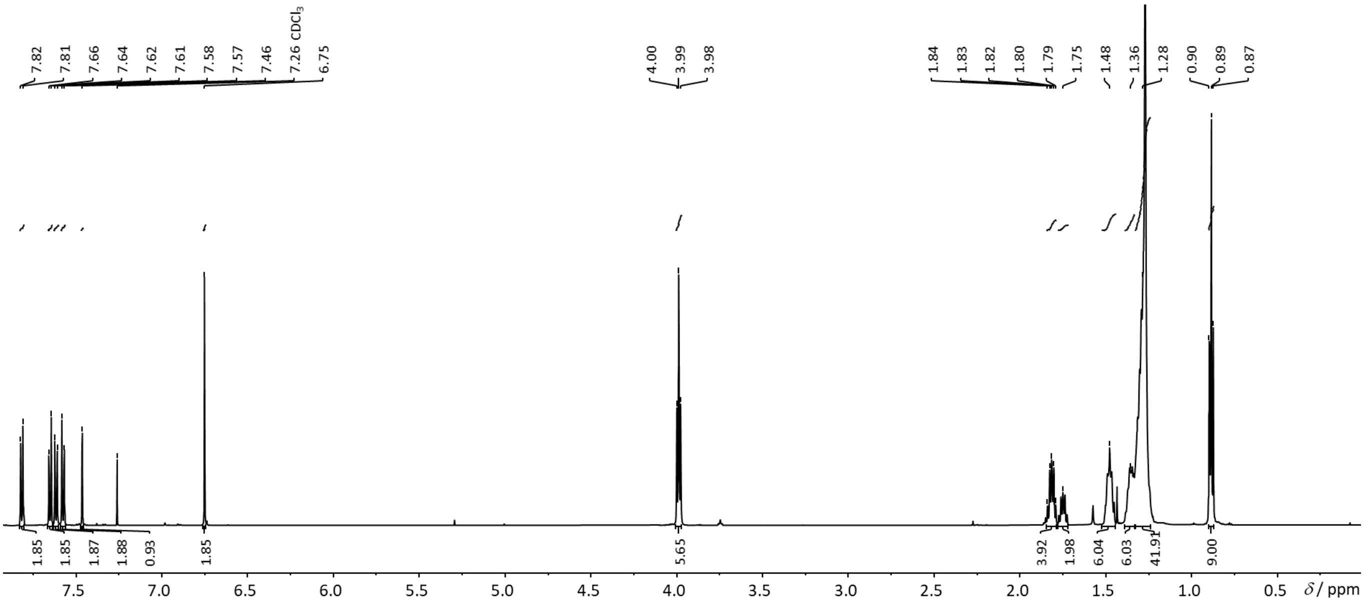

Supplementary Figure 6: ^{1}H-NMR ($CDCl_3$, 600 MHz, 299 K) of (*Z*)-3-(4-iodophenyl)-2-(4-((3,4,5-tris(dodecyloxy)phenyl)ethynyl)phenyl) acrylonitrile.

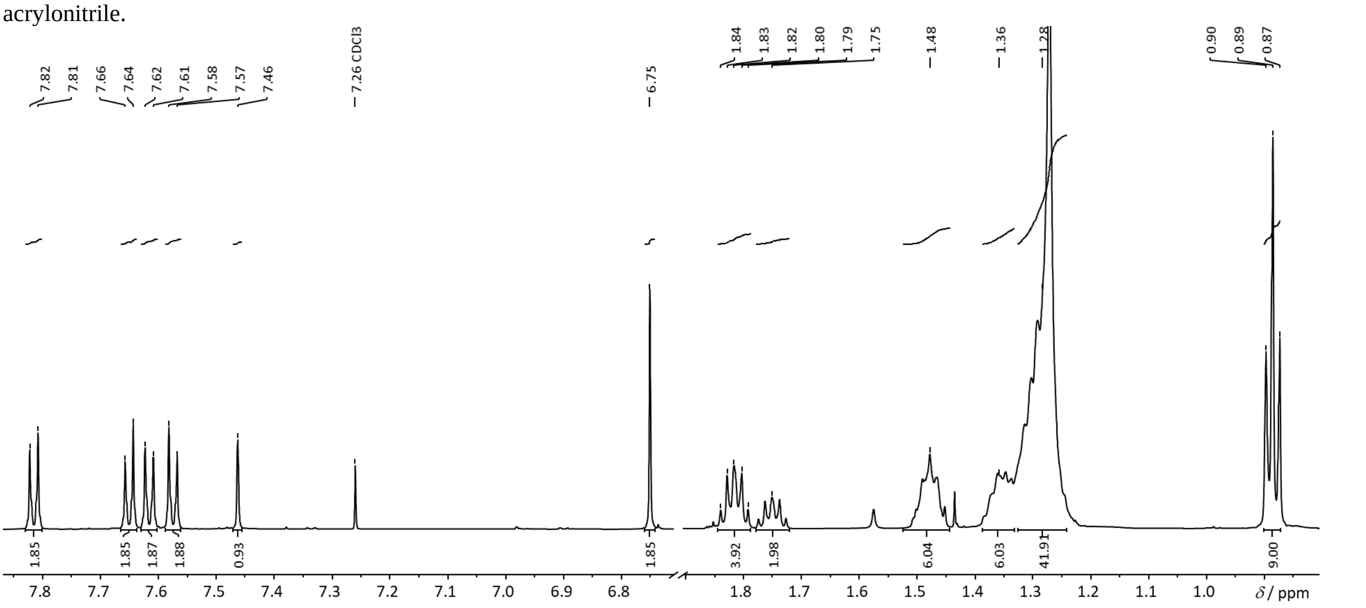

Supplementary Figure 7: Magnification of the aromatic region of the ^{1}H-NMR ($CDCl_3$, 600 MHz, 299 K) of (*Z*)-3-(4-iodophenyl)-2-(4-((3,4,5-tris(dodecyloxy)phenyl)ethynyl)phenyl)acrylonitrile.

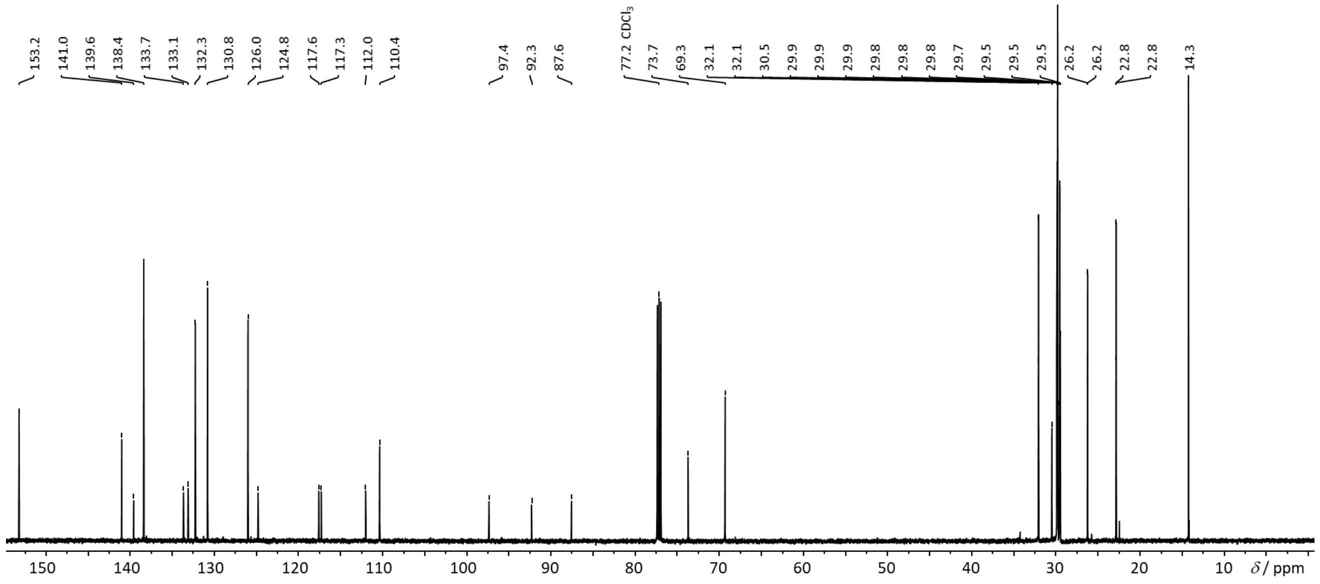

Supplementary Figure 8: ^{13}C-NMR ($CDCl_3$, 151 MHz, 299 K) of (*Z*)-3-(4-iodphenyl)-2-(4-((3,4,5-tris(dodecyloxy)phenyl)ethynyl) phenyl) acrylonitrile.

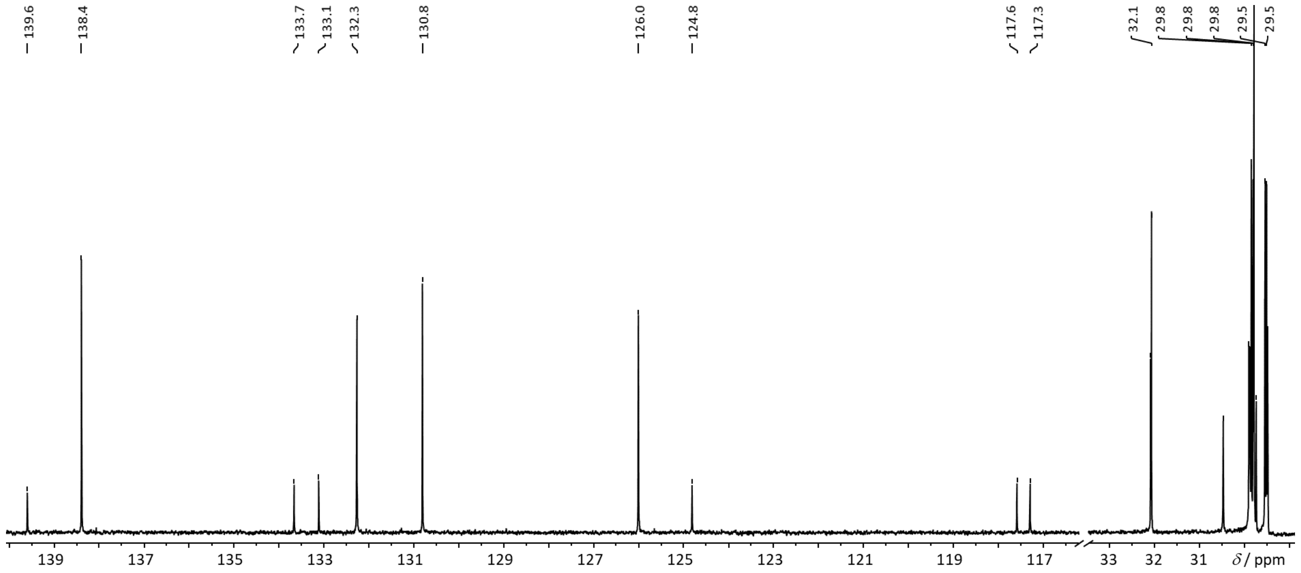

Supplementary Figure 9: Magnification of the aromatic region of the ^{13}C-NMR ($CDCl_3$, 151 MHz, 299 K) of (*Z*)-3-(4-iodphenyl)-2-(4-((3,4,5-tris(dodecyloxy) phenyl)ethynyl)phenyl)acrylonitrile.

5,5'-bis((trimethylsilyl)ethynyl)-2,2'-bipyridine

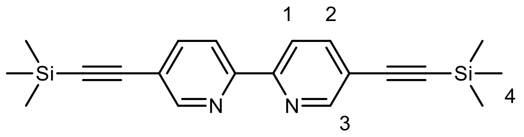

$C_{20}H_{24}N_2Si_2$
348.5960g/mol

Following a modified reaction procedure published in the literature,[20] 5,5'-dibromo-2,2'-bipyridine (1.00 g, 3.19 mmol, 1.00 eq), $Pd(PPh_3)_4$ (134 mg, 6 mol%) and CuI (6 mg, 1 mol%) were dispersed in NEt_3 (20 mL, distilled/degassed) and THF (5 mL, distilled/degassed) and the resulting mixture was stirred at r.t. for 20 min. Trimethylsilylacetylene (1.77 mL, 1.25 g, 12.74 mmol, 4.00 eq) was slowly added dropwise, the reaction mixture

was heated up to 70 °C and subsequently stirred for 14 h. After cooling to r.t., the mixture was diluted with DCM (30 mL) and filtered over Celite.® The solution was washed with NH_3 aq. (25 %, 2×15 mL) and water (10 mL). All volatile compounds were removed under reduced pressure and the residue was purified by column chromatography (SiO_2, 1 % MeOH/DCM). The product was obtained as a colorless solid (1.10 g, 3.16 mmol, 99 %). **MS** (ESI Nanospray, $CH_3CN/CHCl_3$): *m/z* (%) = 349.15473 (100) $[M+H]^+$, 371.13661 (74) $[M+Na]^+$, 719.28445 $[2M+Na]^+$, calculated for: $C_{20}H_{25}N_2Si_2^+$ 349.15508, $C_{20}H_{24}N_2Si_2Na^+$ 371.13702, $C_{40}H_{48}N_4Si_4Na^+$ 719.28482. **^{1}H-NMR** ($CDCl_3$, 400 MHz, 299 K): δ(ppm) = 8.73 (*dd*, *J* = 2.1, 0.9 Hz, 2H, H_3), 8.38 (*dd*, *J* = 8.4, 0.9 Hz, 2H, H_1), 7.87 (*dd*, *J* = 8.4, 2.1 Hz, 2H, H_2), 0.28 (*s*, 18H, H_4). The analytical results are in good agreement with the data published in the literature.[20]

5,5'-diethynyl-2,2'-bipyridine

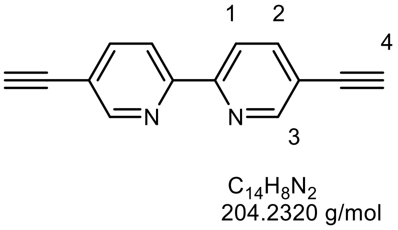

According to a literature procedure[20], 5,5'-bis((trimethylsilyl)ethynyl)-2,2'-bipyridine (200 mg, 0.57 mmol, 1.00 eq) was dissolved in a 1:1 mixture of MeOH and THF (8 mL). K_2CO_3 (397 mg, 2.87 mmol, 5.00 eq) was added and the reaction mixture was stirred at r.t. for 3 h. The mixture was poured on water (10 mL) and subsequently extracted with DCM (3×10 mL). The combined organic layers were dried over $MgSO_4$ and all volatile compounds were removed under reduced pressure. The desired product was obtained as a colorless crystalline solid without further purification (117 mg, 0.57 mmol, 100 %). **MS** (ESI Nanospray, $CH_3OH/CHCl_3$): *m/z* = 205.07588 $[M+H]^+$, 227.0579 $[M+Na]^+$, calculated for: $C_{14}H_9N_2^+$ 205.07602, $C_{14}H_8N_2Na^+$ 227.05797. **^{1}H-NMR** ($CDCl_3$, 400 MHz, 300 K): δ(ppm) = 8.76 (*dd*, *J* = 2.2, 0.9 Hz, 2H, H_3), 8.39 (*dd*, *J* = 8.2, 0.9 Hz, 2H, H_1), 7.90 (*dd*, *J* = 8.2, 2.1 Hz, 2H, H_2), 3.30 (*s*, 2H, H_4). The analytical results are in good agreement with the data published in the literature.[20]

(2*Z*,2'*Z*)-3,3'-(([2,2'-bipyridine]-5,5'-diylbis(ethyne-2,1-diyl))bis(4,1-phenylene))bis(2-(4-((3,4,5-tris(dodecyloxy)phenyl)ethynyl)phenyl)acrylonitrile) [(Z)1]

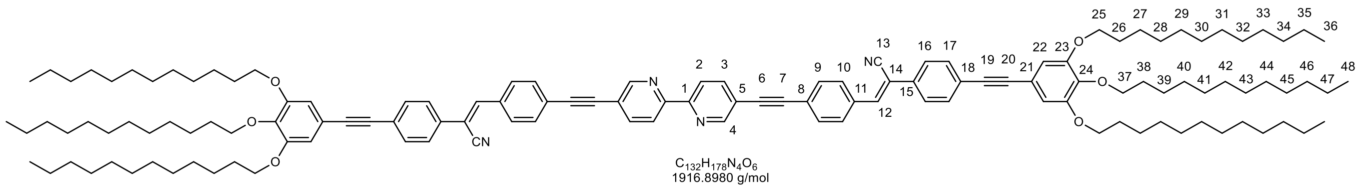

(*Z*)-3-(4-iodophenyl)-2-(4-((3,4,5-tris(dodecyloxy)phenyl)ethynyl)phenyl)acrylonitrile (700 mg, 0.711 mmol, 2.00 eq), $Pd(PPh_3)_4$ (29 mg, 7 mol%) and CuI (3 mg, 2 mol%) were dispersed in NEt_3 (8 mL, distilled/degassed) and THF (8 mL, distilled/degassed) and the resulting mixture was stirred at r.t. for 20 min. 5,5'-diethynyl-2,2'-bipyridine

(73 mg, 0.356 mmol, 1.00 eq) in THF (3 mL, distilled/degassed) was slowly added dropwise, the reaction mixture was heated up to 50 °C and stirred for 14 h. The reaction was allowed to cool to r.t., diluted with DCM (25 mL) and filtered over Celite.® The solution was washed with an aqueous NH_3 solution (25 %, 25 mL) and the organic layer was dried over $MgSO_4$. All volatile compounds were removed under reduced pressure and the residue was purified by column chromatography (SiO_2, 0-2.5 % MeOH/DCM). The desired product was obtained as an intense orange-colored solid (580 mg, 0.303 mmol, 85 %). **MS** (ESI Nanospray, $CHCl_3/CH_3CN$): *m*/*z* = 1939.37035 $[M+Na]^+$, calc. for: $C_{132}H_{178}N_4O_6Na$ 1939.36724, $C_{142}H_{188}N_6O_{12}$ 1037.23063. **^{1}H-NMR** (THF-d_8, 600 MHz, 299 K), δ(ppm) = 8.82 (*d*, J = 2.1 Hz, 2H, H_4), 8.55 (*d*, J = 8.2 Hz, 2H, H_2), 8.05 – 8.01 (*m*, 6H, H_3+H_{10}), 7.86 (*s*, 2H, H_{12}), 7.79 (*d*, J = 8.1 Hz, 4H, H_{16}), 7.70 (*d*, J = 8.1 Hz, 4H, H_9), 7.59 (*d*, J = 8.0 Hz, 4H, H_{17}), 6.80 (*s*, 4H, H_{22}), 3.99 (*t*, J = 6.4 Hz, 8H, H_{25}), 3.96 (*t*, J = 6.3 Hz, 4H, H_{37}), 1.84 - 1.78 (*m*, 8H, H_{26}), 1.76 - 1.70 (*m*, 4H, H_{38}) (overlaps with THF-d_8), 1.56 - 1.50 (*m*, 12H, H_{27}+H_{39}), 1.44 - 1.28 (*m*, 96H, H_{28-35}+H_{40-47}), 0.96 - 0.86 (*m*, 18H, H_{36}+H_{48}). **^{13}C-NMR** (CD_2Cl_2, 151 MHz, 299 K): δ(ppm) = 155.4 (C_1), 154.3 (C_{23}), 152.8 (C_4), 141.9 (C_{12}), 140.9 (C_{24}), 140.3 (C_2), 135.6 (C_{11}), 135.1 (C_{15}), 133.1 (C_9), 132.3 (C_{17}), 130.6 (C_{10}), 127.0 (C_{16}), 125.8 (C_8), 121.5 (C_3), 121.3 (C_5), 118.3 (C_{21}), 118.2 (C_{13}), 112.7 (C_{14}), 111.3 (C_{22}), 94.2 (C_7), 93.2 (C_{20}), 89.8 (C_6), 88.2 (C_{19}), 73.9 (C_{37}), 70.0 (C_{25}), 33.1 (-CH_2-), 33.1 (-CH_2-), 31.6 (C_{38}), 31.0 (-CH_2-), 30.9 (-CH_2-), 30.9 (-CH_2-), 30.9 (-CH_2-), 30.8 (-CH_2-), 30.8 (-CH_2-), 30.8(-CH_2-), 30.6 (-CH_2-), 30.3 (-CH_2-), 30.5 (-CH_2-), 30.5 (-CH_2-), 27.3 (C_{39}), 27.3 (C_{27}), 23.8 (-CH_2-), 23.7 (-CH_2-), 14.6 (C_{36}+C_{48}). To characterize the configuration of the central C=C-bond, the C_{12}-H_{10} coupling constant was determined. The coupling constant was found to be $^3J_{CN\text{-}H}$ = 14.7 Hz, which is in good agreement with the corresponding value for *Z*-configured CN-substituted stilbenes reported in the literature.

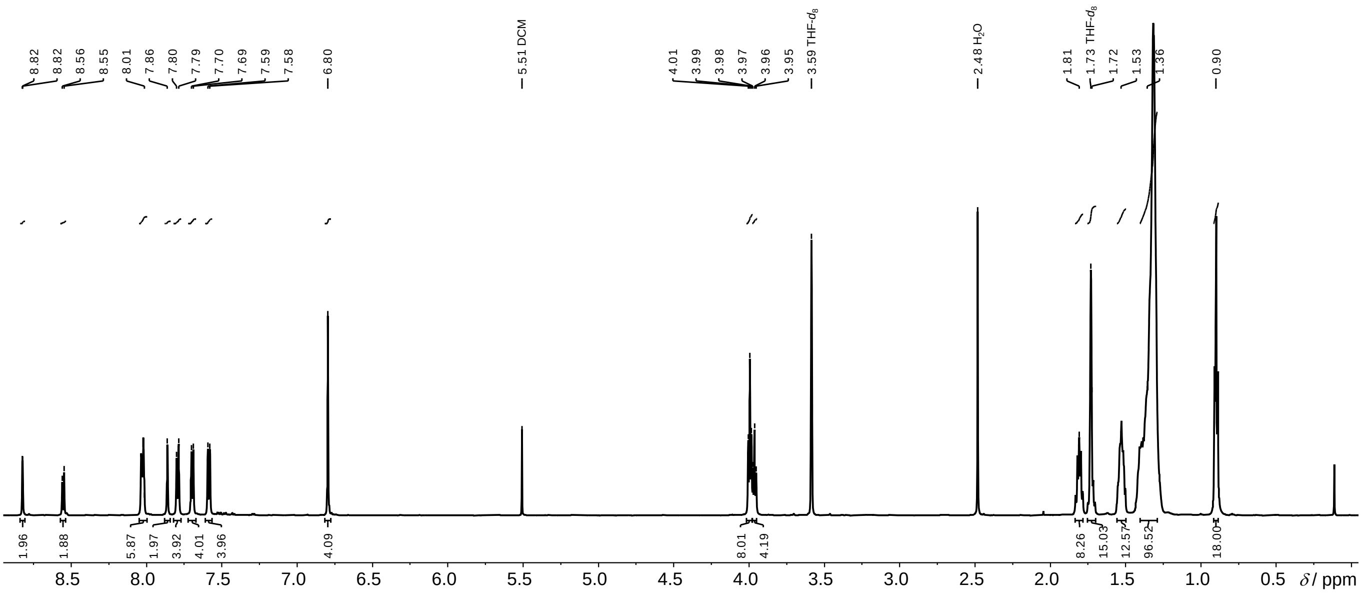

Supplementary Figure 10: ^{1}H-NMR (THF-d_8, 600 MHz, 299 K) of (Z)**1**.

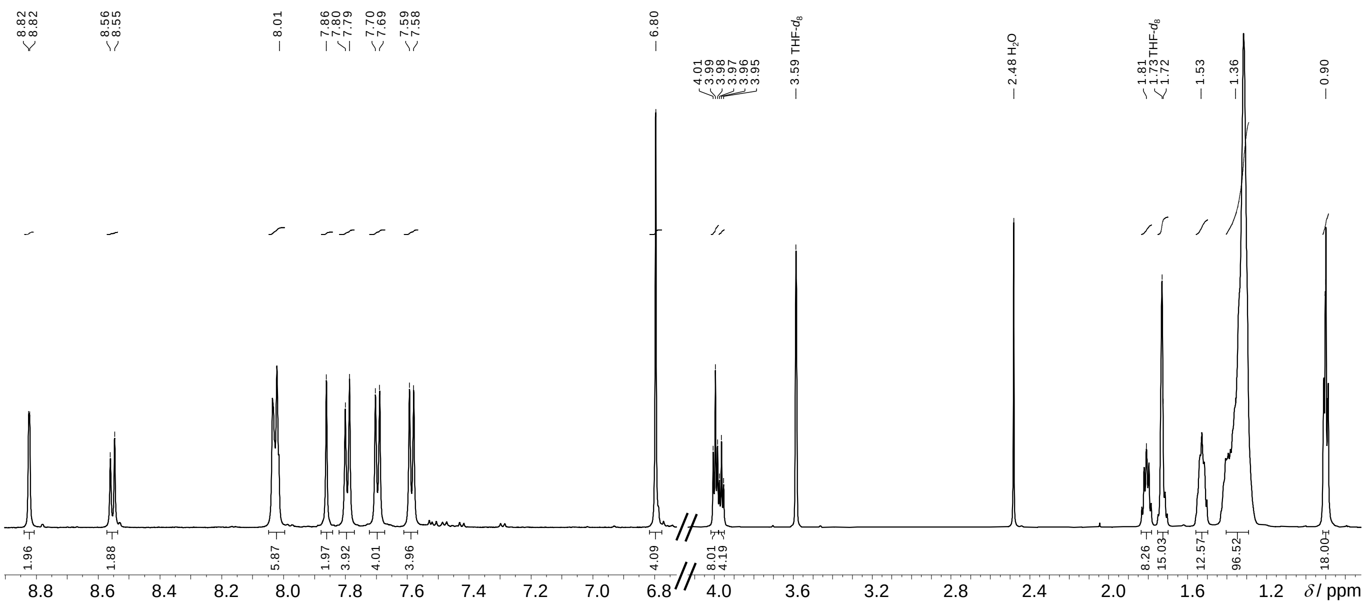

Supplementary Figure 11: Magnification of the aromatic region of the ^{1}H-NMR (THF-d_8, 600 MHz, 299 K) of (Z)**1**.

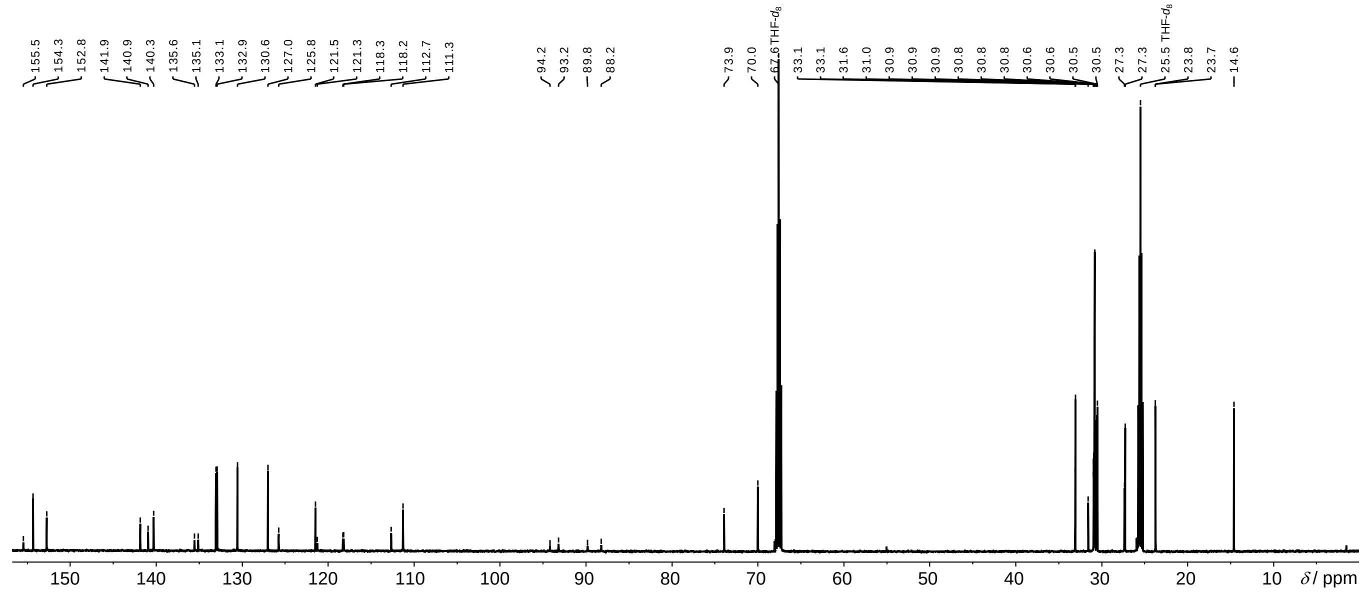

Supplementary Figure 12: ^{13}C-NMR (THF-d_8, 151 MHz, 299 K) of (Z)**1**.

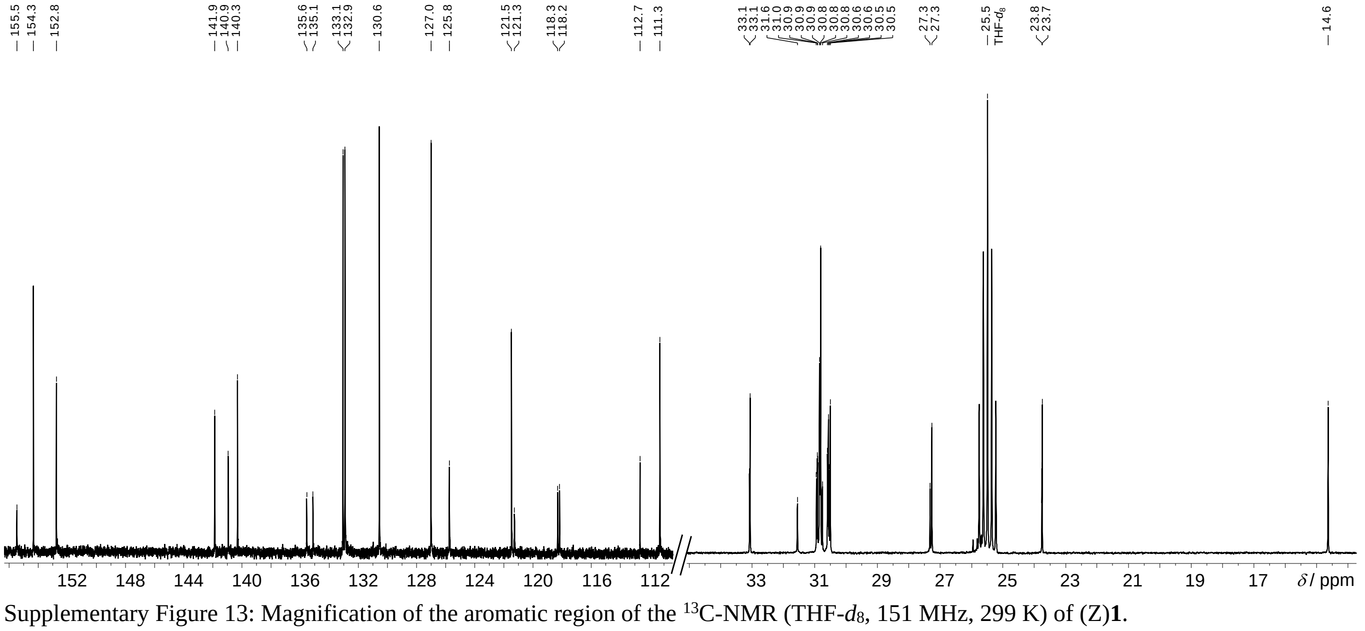

Supplementary Figure 13: Magnification of the aromatic region of the ^{13}C-NMR (THF-d_8, 151 MHz, 299 K) of (Z)**1**.

2 Nucleation-Elongation model for Cooperative Supramolecular Polymerizations

The equilibrium between the monomeric and supramolecular species can be described in a cooperative process with the *nucleation-elongation model* which is developed by Ten Eikelder, Markvoort and Meijer.[21,22] This model is used to describe the aggregation of (Z)**1** which exhibits non-sigmoidal cooling curves as shown in fluorescence and UV-Vis temperature-dependent experiments. The model extends nucleation-elongation based equilibrium models for growth of supramolecular homopolymers to the case of two monomer and aggregate types and can be applied to symmetric supramolecular copolymerizations, as well as to the more general case of nonsymmetric supramolecular copolymerizations.

In a cooperative process, the polymerization occurs by a nucleation step, to a nucleus size assumed of B, and a following elongation step. The values T_e, $\Delta H°_{nucl}$, $\Delta H°$ and $\Delta S°$ can be determined by a non-linear least-square analysis of the experimental melting curves. The equilibrium constants associated with the nucleation and elongation phases can be calculated using equations 1 and 2:

$$\text{Nucleation step: } K_n = e^{\left(\frac{-(\Delta H^o - \Delta H^o_{\mathrm{nucl}}) - T\Delta S^o}{RT}\right)} \qquad (1)$$

$$\text{Elongation step: } K = e^{\left(\frac{-(\Delta H^o - T\Delta S^o)}{RT}\right)} \qquad (2)$$

And the cooperativity factor (σ) is given by:

$$\sigma = \frac{K_n}{K_e} = e^{\left(\frac{\Delta H^o_{\mathrm{nucl}}}{RT}\right)} \qquad (3)$$

3 Supplementary Figures

3.1 Solvent-dependent spectroscopic behavior

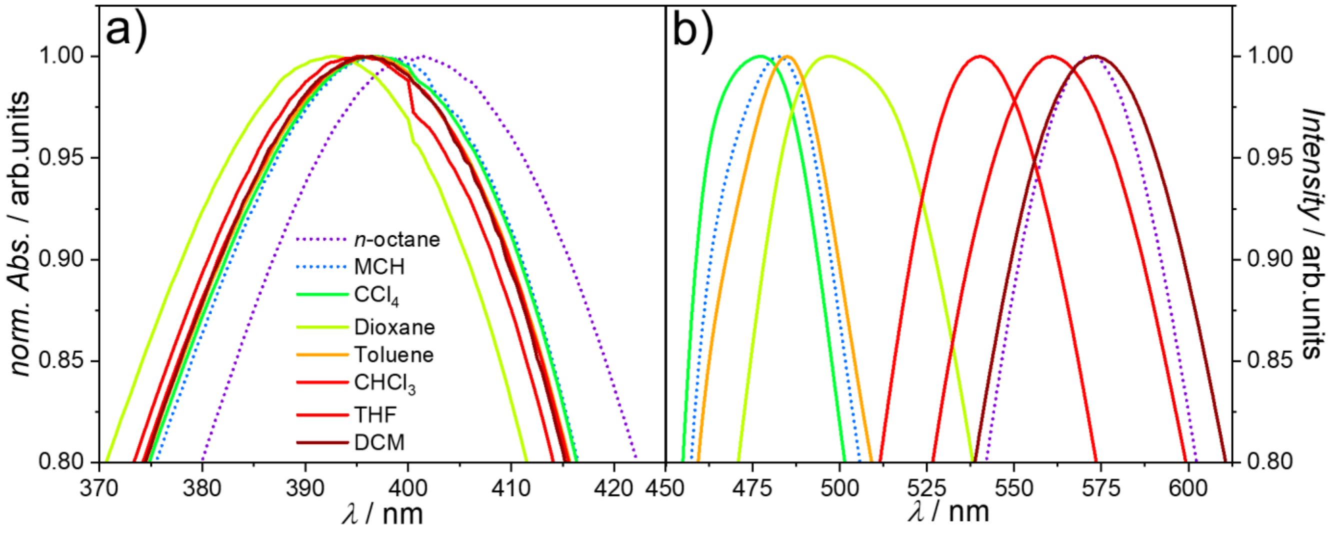

Supplementary Figure 14: Solvatochromic behavior of (Z)**1**. a) Normalized UV-Vis-spectra of (Z)**1** (1×10^{-5} M, 298 K) in different organic solvents in the molecularly dissolved (solid lines) and aggregated (dotted lines) state. b) Corresponding normalized emission spectra excited at λ_{max} of the respective absorbance.

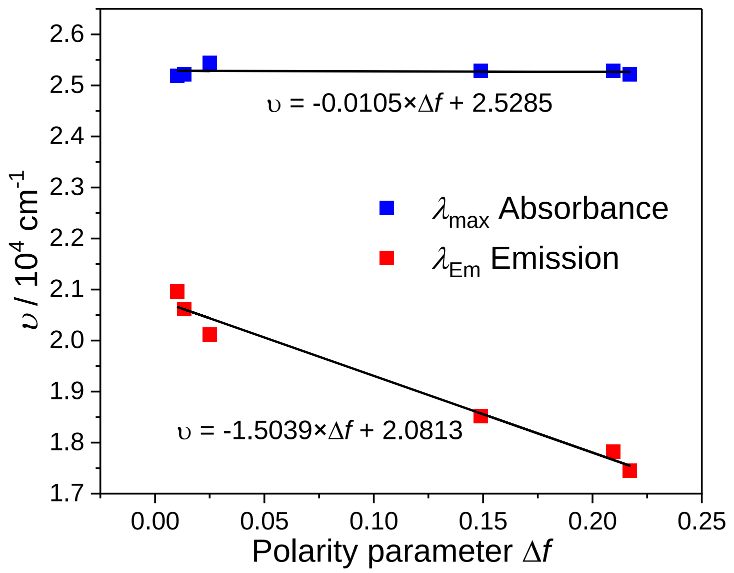

Supplementary Figure 15: Lippert-Mataga plot[23,24] of (Z)**1**. The Lippert-Mataga plot is used to display the dependency of different excited states by plotting the respective wavenumber of λ_{max} and λ_{Em} against the polarity parameter, which was obtained using the equation[23] $\Delta f = f(e\varepsilon) - f(n^2) = \frac{\varepsilon-1}{2\varepsilon-1} - \frac{n^2}{2n^2-1}$.

Analysis *via* a Lippert-Mataga-plot[24] reveals a more polar excited state than the ground state (Supplementary Figure 15). The observed effects can be attributed to either solvent stabilization of the excited state and/or access to ICT or TICT states.[25–27]

3.2 Stimuli-responsive behavior

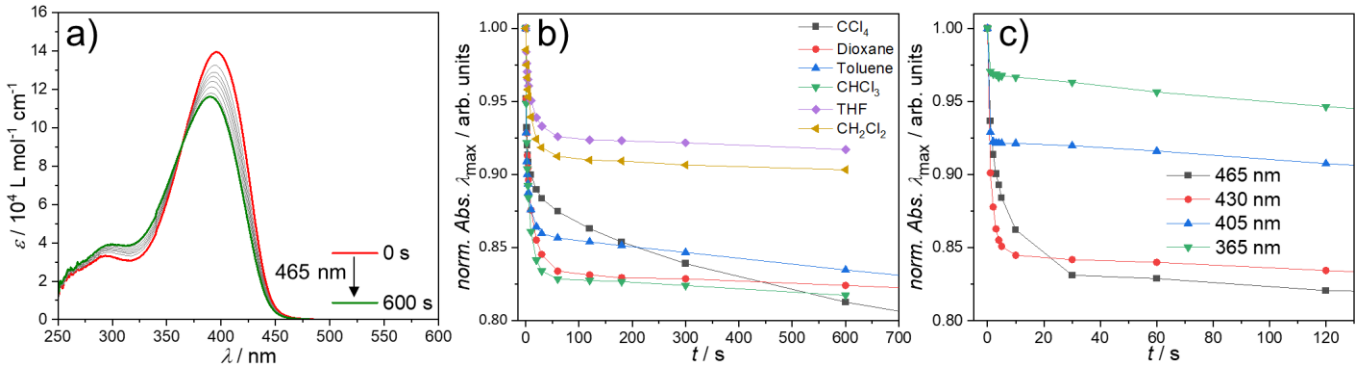

Supplementary Figure 16: Summary of the light-responsive behavior of (Z)**1**. a) UV-Vis spectra of (Z)**1** (1×10^{-5} M, $CHCl_3$, 298 K) under irradiation with λ_{LED} = 465 nm. b) Evolution of absorbance at λ_{max} over irradiation time (λ_{LED} = 465 nm) in different solvents. c) Evolution of the absorbance at λ_{max} over the irradiation time with different wavelength.

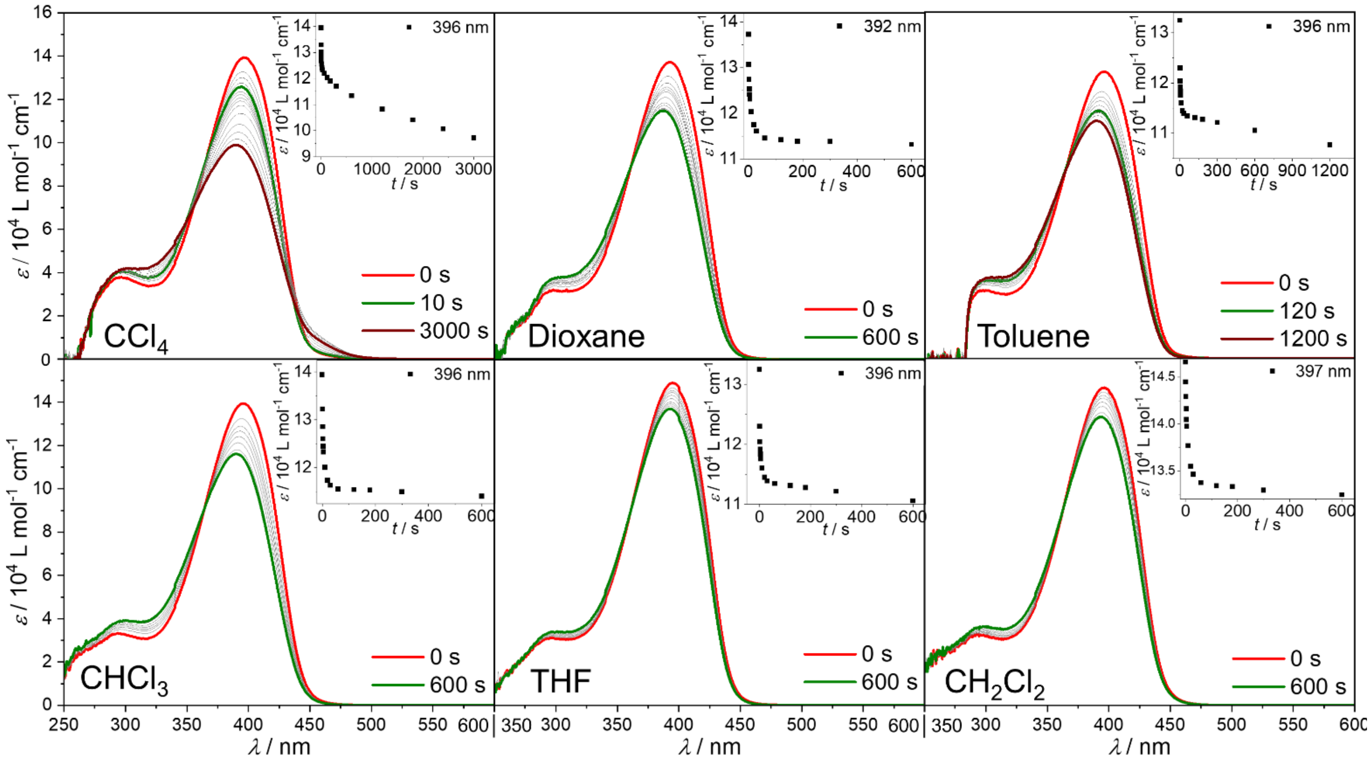

Supplementary Figure 17: Long-term light-response of (Z)**1** in different organic solvents. UV-Vis-spectra of (Z)**1** (1×10^{-5} M, 298 K) under irradiation with λ_{LED} = 465 nm in various solvents. Insets: Plots of the extinction coefficients ε at λ_{max} vs. irradiation time.

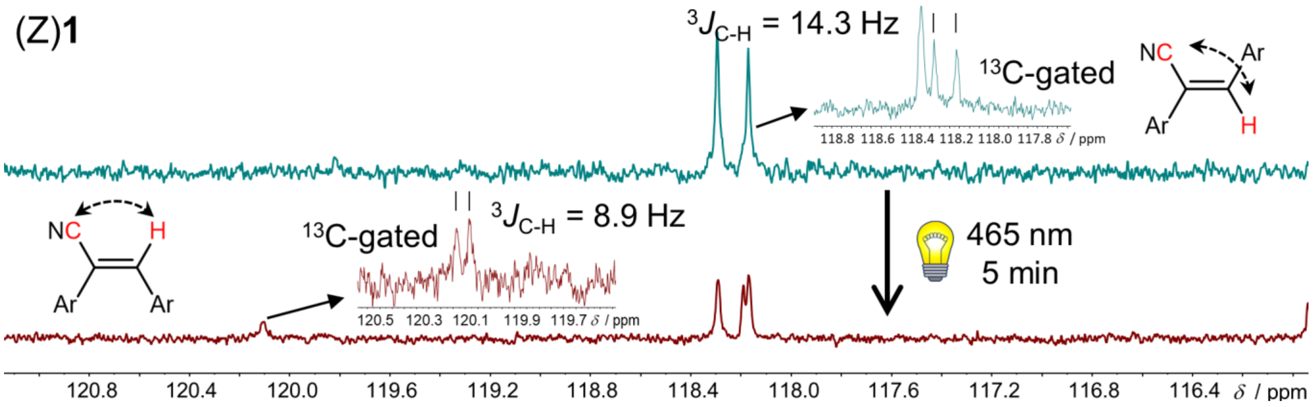

Supplementary Figure 18: Identification of the Z/E-photoisomerization via ^{13}C-gated-NMR spectroscopy. Section of the nitrile-carbon signal region of the ^{13}C-NMR spectra of (Z)**1** (THF-d_8, 151 MHz, 298 K) before (top) and after (bottom) irradiation with λ_{LED} = 465 nm. Insets: gated decoupling ^{13}C-NMR spectra of these signals including their coupling constants.

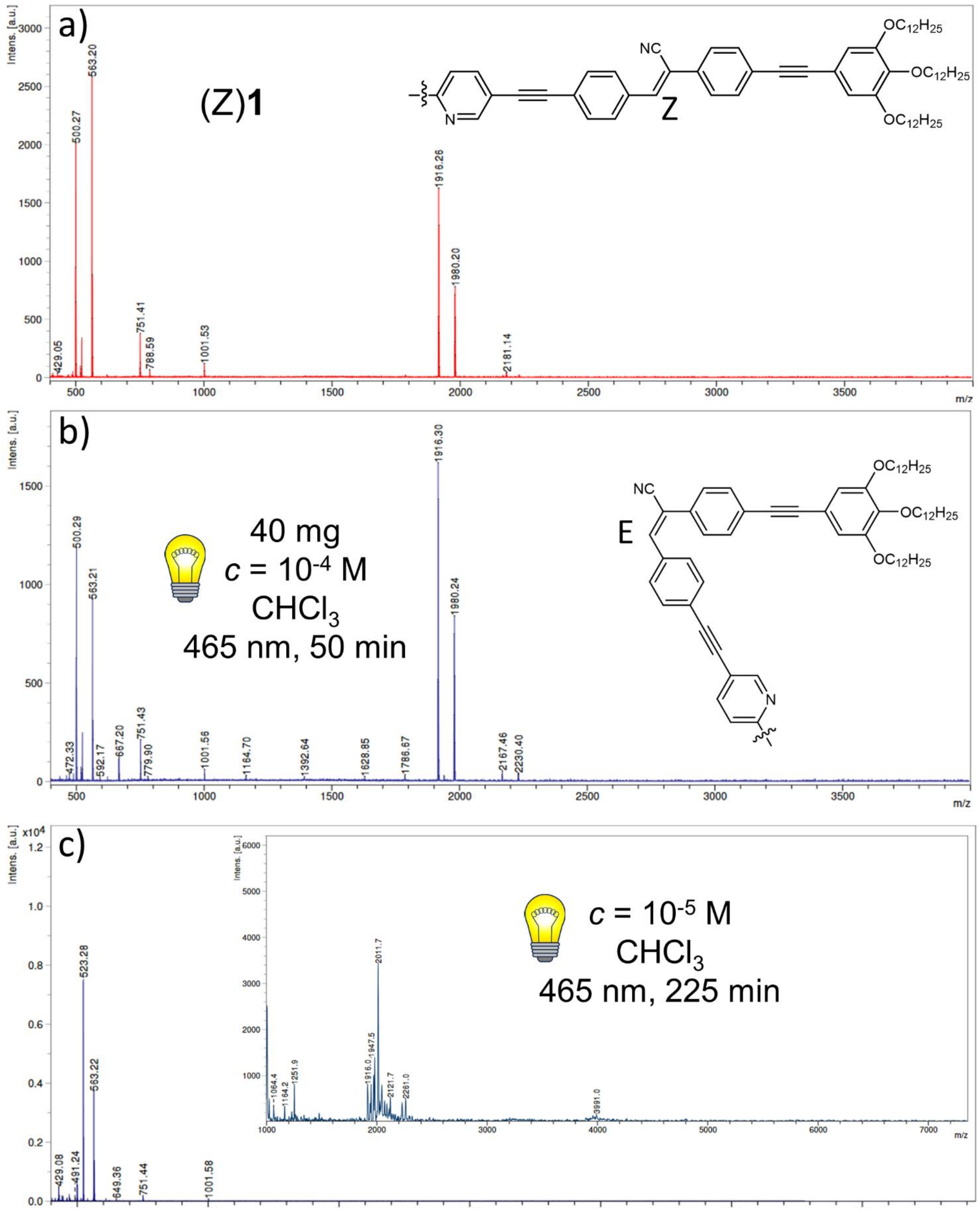

Supplementary Figure 19: Mass spectra of (Z)**1** and its photoproducts. MALDI-TOF spectra (DCTB/$CHCl_3$) of (Z)**1** a) before irradiation, b) after irradiation with λ_{LED} = 465 nm and c) after irradiation with λ_{LED} = 430 nm.

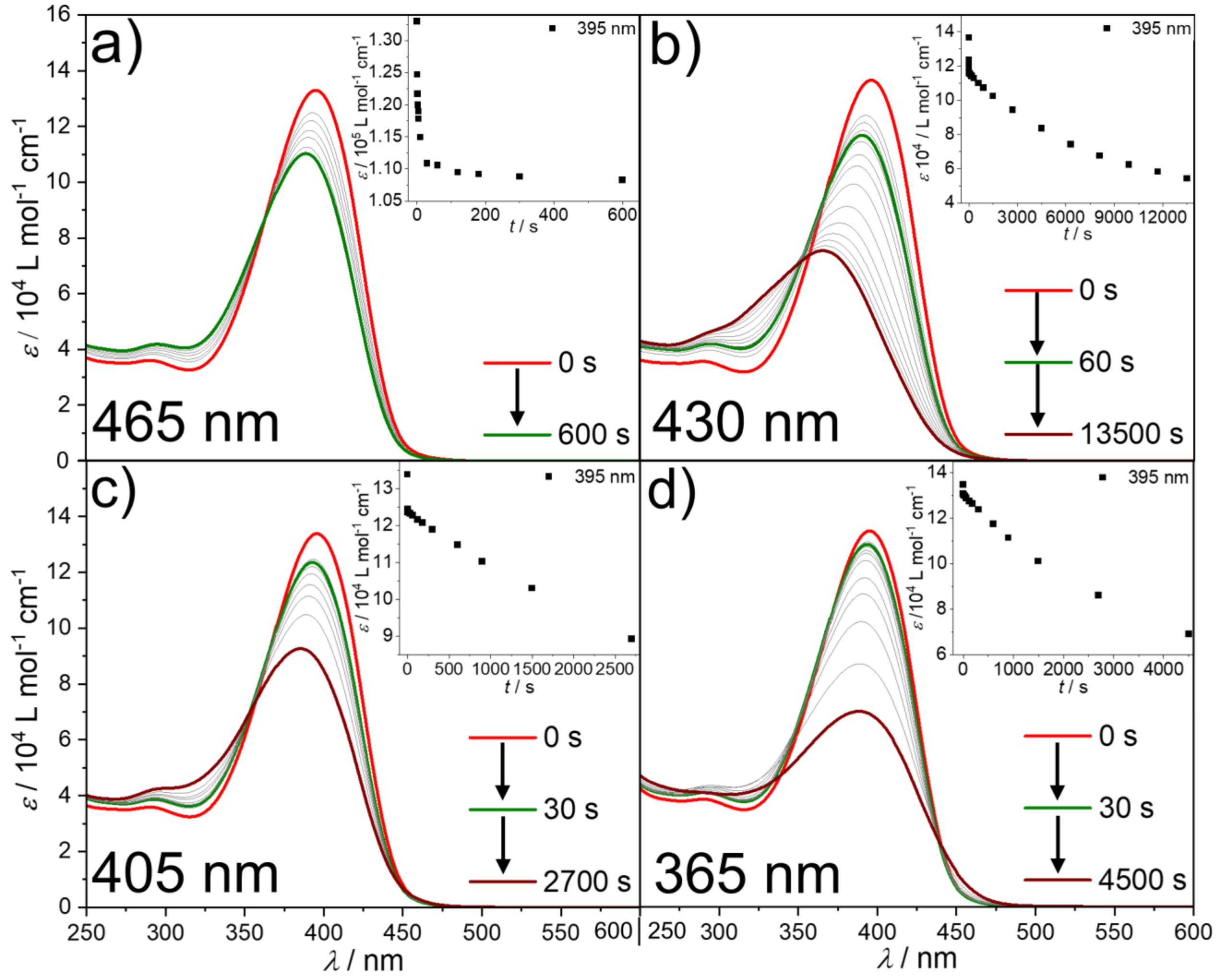

Supplementary Figure 20: Long-term light-response of (Z)**1** upon irradiation with different energies. UV-Vis-spectra of (Z)**1** (1×10^{-5} M, $CHCl_3$, 298 K) under irradiation with a) λ_{LED} = 465 nm, b) λ_{LED} = 430 nm, c) λ_{LED} = 405 nm and d) λ_{LED} = 365 nm. Insets: Plot of the extinction coefficient ε vs. irradiation time.

Using LEDs of higher energy (430-365 nm) than 465 nm and prolonged irradiation times leads to side photoreactions other than Z/E-photoisomerizations. The irradiation studies were carried out in $CHCl_3$ (1.0×10^{-5} M). Overall, two different reactions can be observed using LEDs with energy of 430 nm or higher. For short irradiation times (≤ 120 s), only the expected photoisomerization occurs as discussed above, while for longer times (up to 225 min) a second reaction sets in with λ_{LED} = 430, 405 and 365 nm (Supplementary Figure 20b-d). These are characterized by the loss of the isosbestic point, a significant decrease in the overall absorbance and a subsequent shift of λ_{max} to higher energies. As the first reaction step is assigned to the Z/E-Isomerization, a comparison of all spectra reveals that the most efficient reaction is taking place with λ_{LED} = 465 nm (Supplementary Figure 20a).

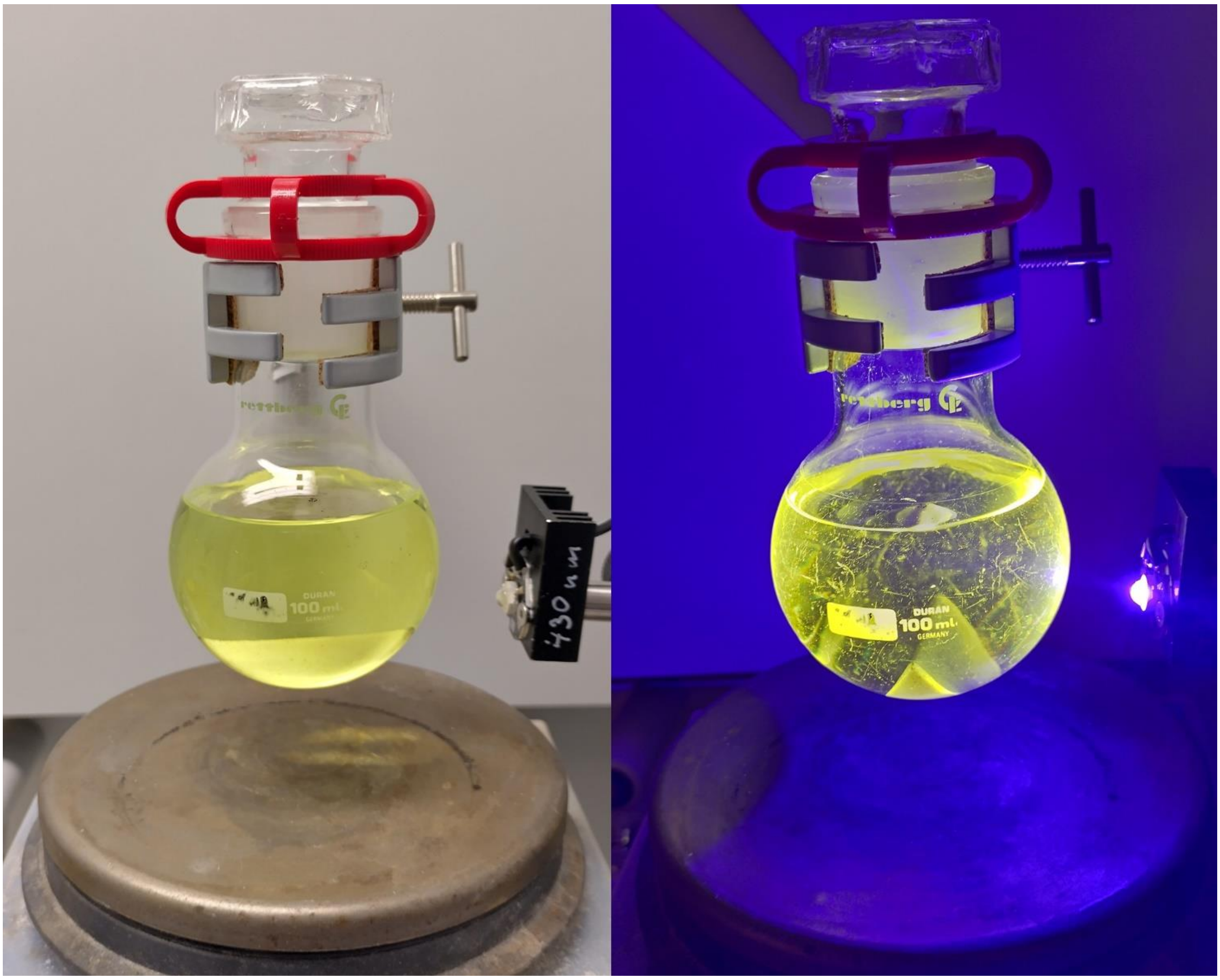

Supplementary Figure 21: Experimental setup of large-scale irradiation experiments.

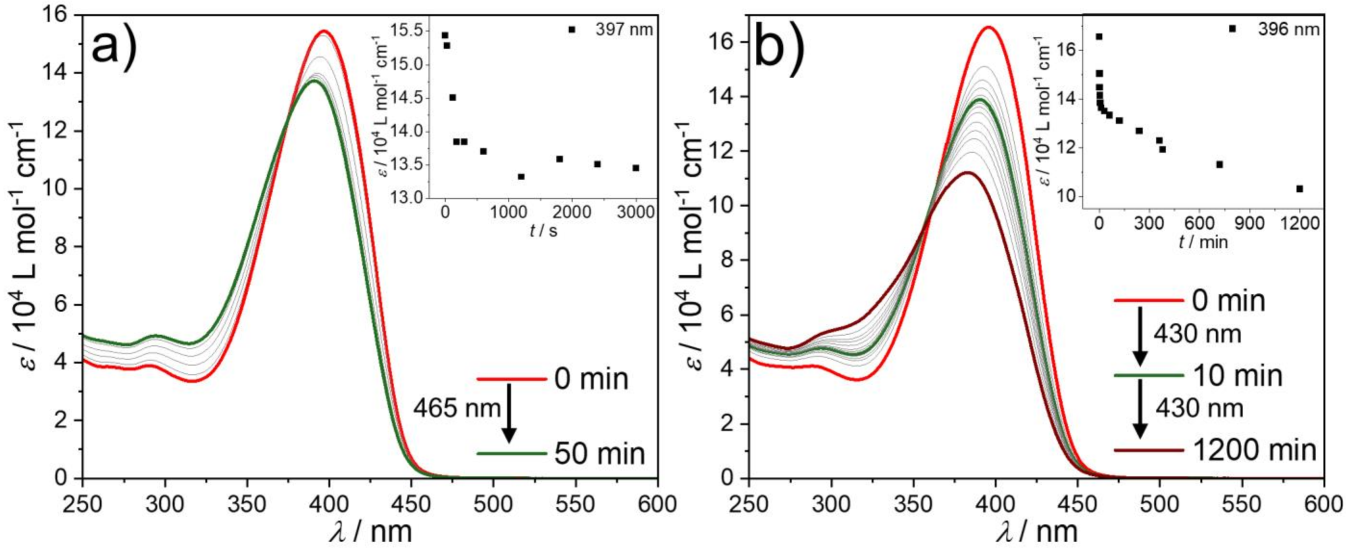

Supplementary Figure 22: Large-scale photoreactions of (Z)**1**. UV-Vis spectra of (Z)**1** (1×10^{-4} M, $CHCl_3$, 298 K) under irradiation with a) λ_{LED} = 465 nm, b) λ_{LED} = 430 nm. Insets: Plot of extinction coefficient ε at λ_{max} versus irradiation time.

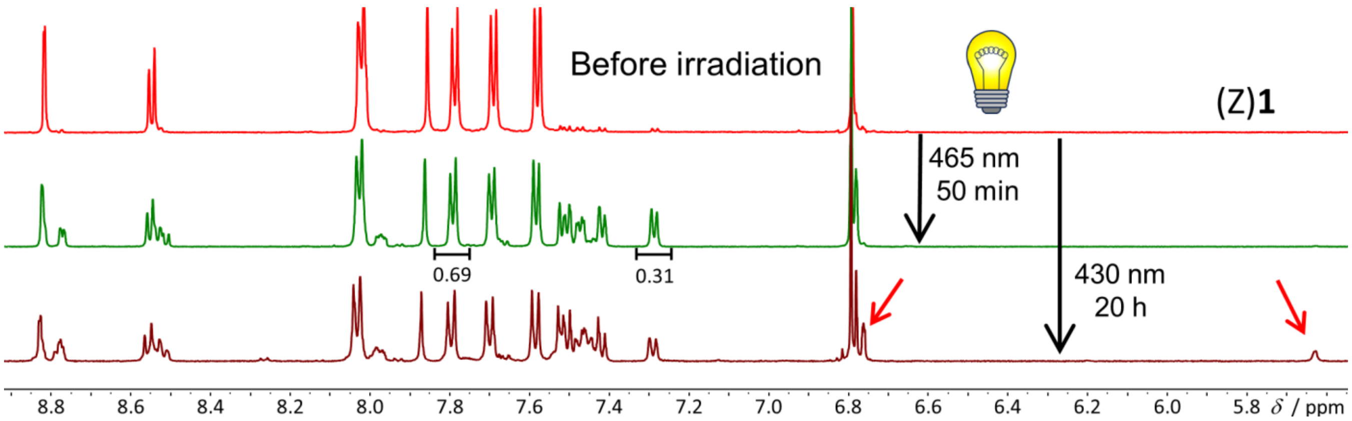

Supplementary Figure 23: Identification of the obtained photoproducts of (Z)**1**. Sections from the aromatic region of the ^{1}H-NMR spectra of (Z)**1** (THF-d_8, 600 MHz, 299 K) before irradiation (red spectrum), after irradiation with λ_{LED} = 465 nm ($CHCl_3$, 1×10^{-4} M, 298 K, 50 min, green spectrum), and after irradiation with λ_{LED} = 430 nm ($CHCl_3$, 1×10^{-4} M, 298 K, 20 h, brown spectrum).

In order to identify the possible photoproducts, the experiments using λ_{LED} = 465 and 430 nm were repeated using higher concentration (1×10^{-4} M) on a larger scale (20 mg) (Supplementary Figure 21). Due to these experimental conditions, the irradiation time increased heavily and both reactions were monitored by UV-Vis-spectroscopy (Supplementary Figure 22). After solvent evaporation, the remaining material could be analyzed by NMR spectroscopy (Supplementary Figure 23). For a λ_{LED} = 465 nm, a set of new highfield shifted proton signals in the aromatic region of the ^{1}H-NMR spectrum can be observed, which resemble the typical pattern of the E-Isomer (Supplementary Figure 23, middle). For λ_{LED} = 430 nm, additional proton signals with unassignable coupling patterns at δ = 6.76 ppm (*t*, *J* = 1.4 Hz) and δ = 6.76 ppm (*s*) can be detected (Supplementary Figure 23, bottom). Additionally, the preexisting patterns in the aromatic region become distorted. A MALDI-TOF spectrum of this reaction showed no sign of higher molecular weights or the Z/E-Isomers (Supplementary Figure 19c). Therefore, it can be assumed that a non-directional side reaction is taking place at long irradiation energies. In summary, the best efficiency for the photoisomerization can be found with λ_{LED} = 465 nm in $CHCl_3$.

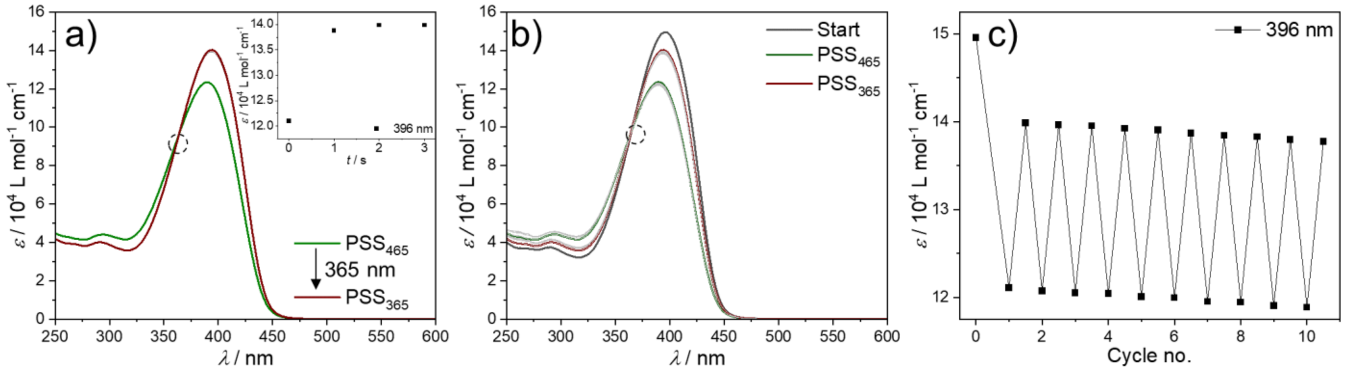

Supplementary Figure 24: Alternate photoswitching and cycling stability of **1** using different wavelengths. a) UV-Vis spectra of (E)**1** (1×10^{-5} M, $CHCl_3$, 298 K) under light irradiation with λ_{LED} = 365 nm. Inset: Evolution of ε at λ = 396 nm over irradiation time. b) All UV-Vis spectra starting from pure (Z)**1** switching back and forth between PSS_{465} and PSS_{365} over 10 consecutive cycles. c) evolution of λ_{max} = 396 nm over the number of applied irradiation cycles.

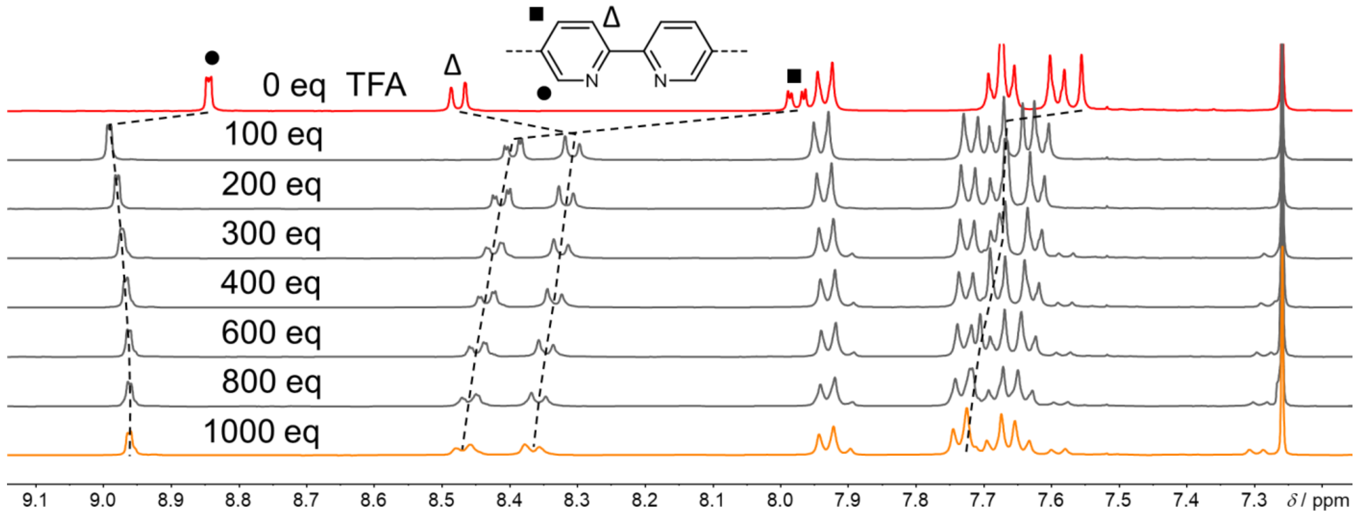

Supplementary Figure 25: Protonation of (Z)**1** by NMR. Sections from the aromatic region of the ^{1}H-NMR spectra of (Z)**1** (4.5×10^{-3} M, $CDCl_3$, 400 MHz, 298 K) under successive addition of TFA-d_1.

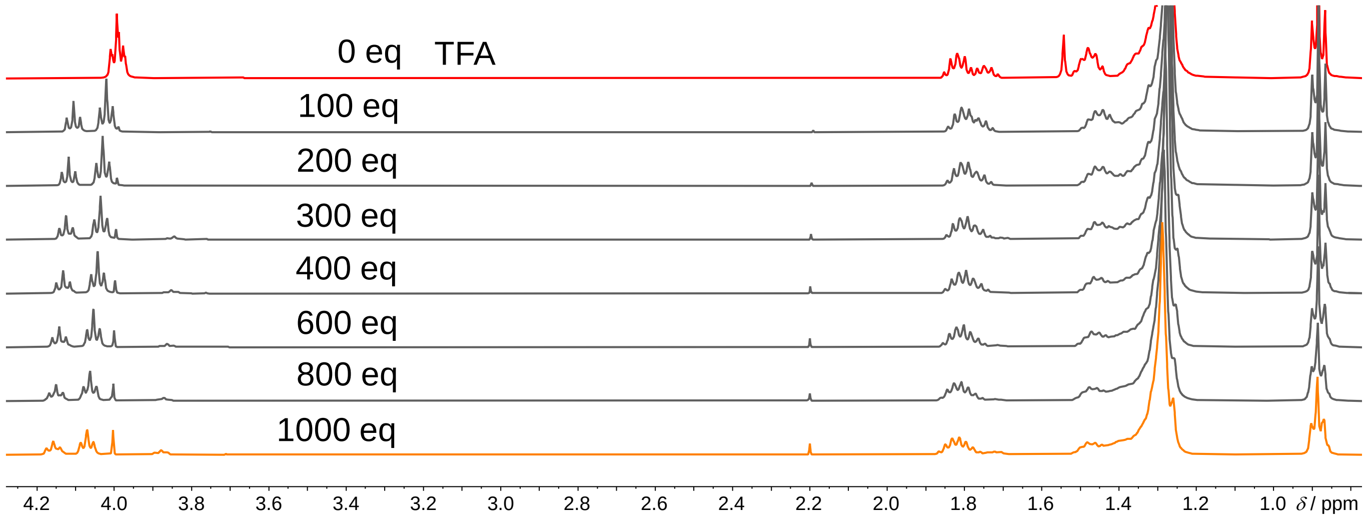

Supplementary Figure 26: Protonation of (Z)**1** by NMR. Sections of the aliphatic region of the ^{1}H NMR spectra ($CDCl_3$, 400 MHz, 298 K) of (Z)**1** under successive addition of TFA-d_1.

For a more detailed view of the protonation at the molecular level, ^{1}H NMR titration studies of (Z)**1** against TFA-d_1 were carried out (4.5×10^{-3} M, $CDCl_3$). In the aromatic region, the most significant changes can be detected for the BPy protons, which undergo remarkable chemical shifts (up to $\Delta\delta = 0.42$ ppm) upon TFA addition, while the remaining proton signals of the aromatic system remain nearly unaffected (Supplementary Figure 25). Only the vinylic proton shows a minor downfield shift ($\Delta\delta = 0.16$ ppm), which might be caused either by dipolar interactions with the acid and/or the neighboring cyano group. In the aliphatic region, only the splitting of the inner and outer alkoxy signals closest to the oxygen atoms can be observed (Supplementary Figure 26). These findings prove that the protonation takes place at the BPy moiety exclusively. Moreover, besides the change in chemical shifts, no splitting of the aromatic BPy signals were detected. Therefore, the symmetry of the molecule seems to be maintained upon protonation. In the literature, examples can be found in which the proton is localized between both nitrogen atoms of the BPy moiety, forcing the system into a cis-like conformation. The experimentally observed signal shifts in the NMR studies of the protonated state is in good agreement with those of 4,4'-BPy-based Pt(II) complexes, in which a cis-configuration is locked upon metal complexation.[28]

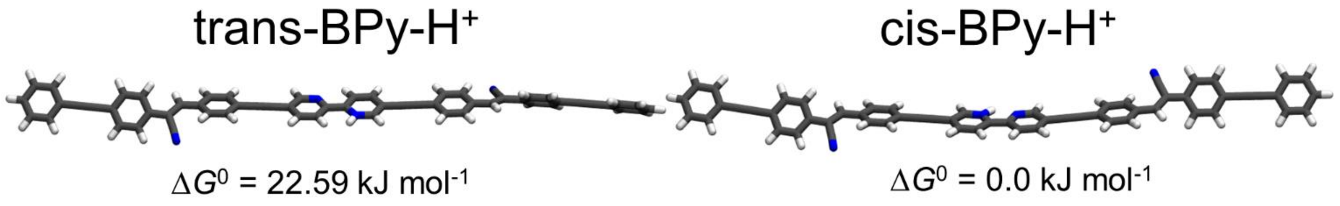

Supplementary Figure 27: Computed trans- and cis- mono-protonated configurations of (Z)**1**. Geometry-optimized [level of theory: GFN2-xTB (6.4.1)] molecular structures of the possible mono-protonated configurations of (Z)**1**. To keep the calculations manageable, the solubility-inducing peripheral dodecyl chains were removed for the calculations due to their chemical inertness to acids.

Supplementary Table 1: Calculated energies of various of the protonated species (Z)**1** in cis- and trans-configuration of the central BPy unit ([$G = E_{tot} + G_{solv} + G_{therm}$; level of theory: E_{tot}: PW6B95-D3/def2-TZVP; G_{solv} (298 K, DCM): BP86/def2-TZVP; G_{therm} (298 K): GFN2-xTB (6.4.1)]).

	E_{tot} / E_h	G_{therm} / E_h	G_{solv} / E_h	G / kcal mol^{-1}	*Diff.* / kcal mol^{-1}
trans	-2530.28961	0.62576	-0.09534	-1587448.1970	5.39820
cis	-2530.30064	0.62667	-0.09382	-1587453.5951	0.00000

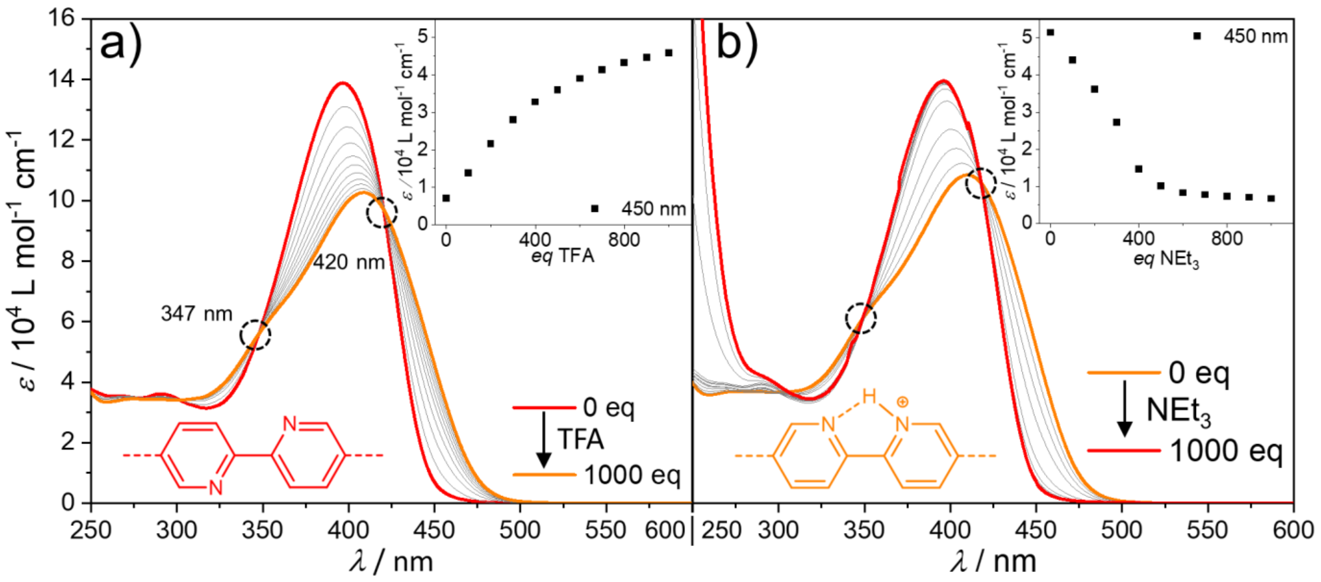

Supplementary Figure 28: Reversible protonation/deprotonation of (Z)**1**. a) UV-Vis spectra of (Z)**1**(1×10^{-5} M, $CHCl_3$, 298 K) under successive addition of TFA. Inset: Plot of the extinction coefficient ε at $\lambda = 450$ nm vs. the added equivalents of TFA. b) UV-Vis spectra of (Z)**1**-H$^+$ (1×10^{-5} M, $CHCl_3$, 298 K) over successive additions of NEt_3. Inset: plot of extinction coefficients at $\lambda = 450$ nm *vs*. equivalents of NEt_3 added.

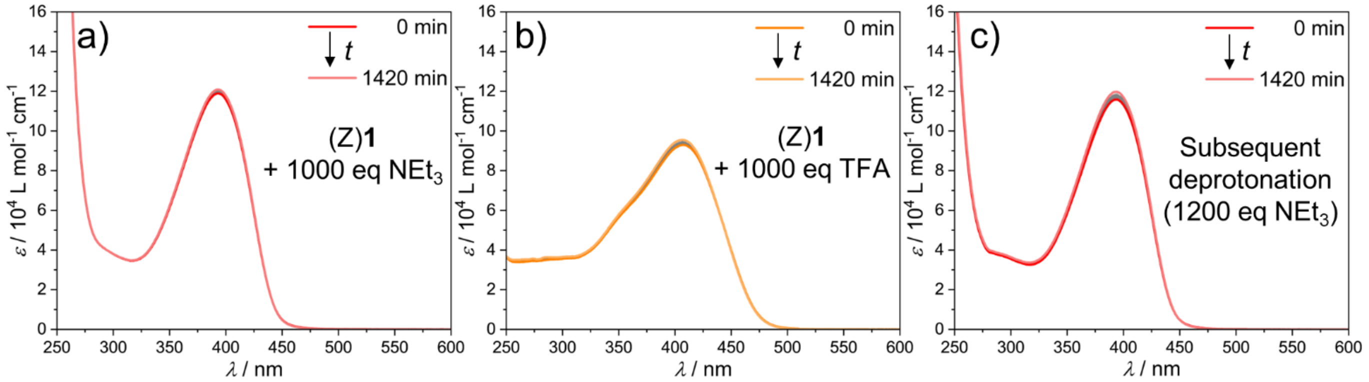

Supplementary Figure 29: Long-term stability of (Z)**1** under basic and acidic conditions. a) Time-dependent UV-Vis spectra of (Z)**1**(1×10^{-5} M, $CHCl_3$, 298 K, 24 h) after addition of 1000 eq NEt_3. b) Time-dependent UV-Vis spectra of (Z)**1**(1×10^{-5} M, $CHCl_3$, 298 K, 24 h) after addition of 1000 eq TFA. c) Time-dependent UV-Vis spectra of (Z)**1** (1×10^{-5} M, $CHCl_3$, 298 K, 24 h) after the deprotonation of b) with 1200 eq NEt_3.

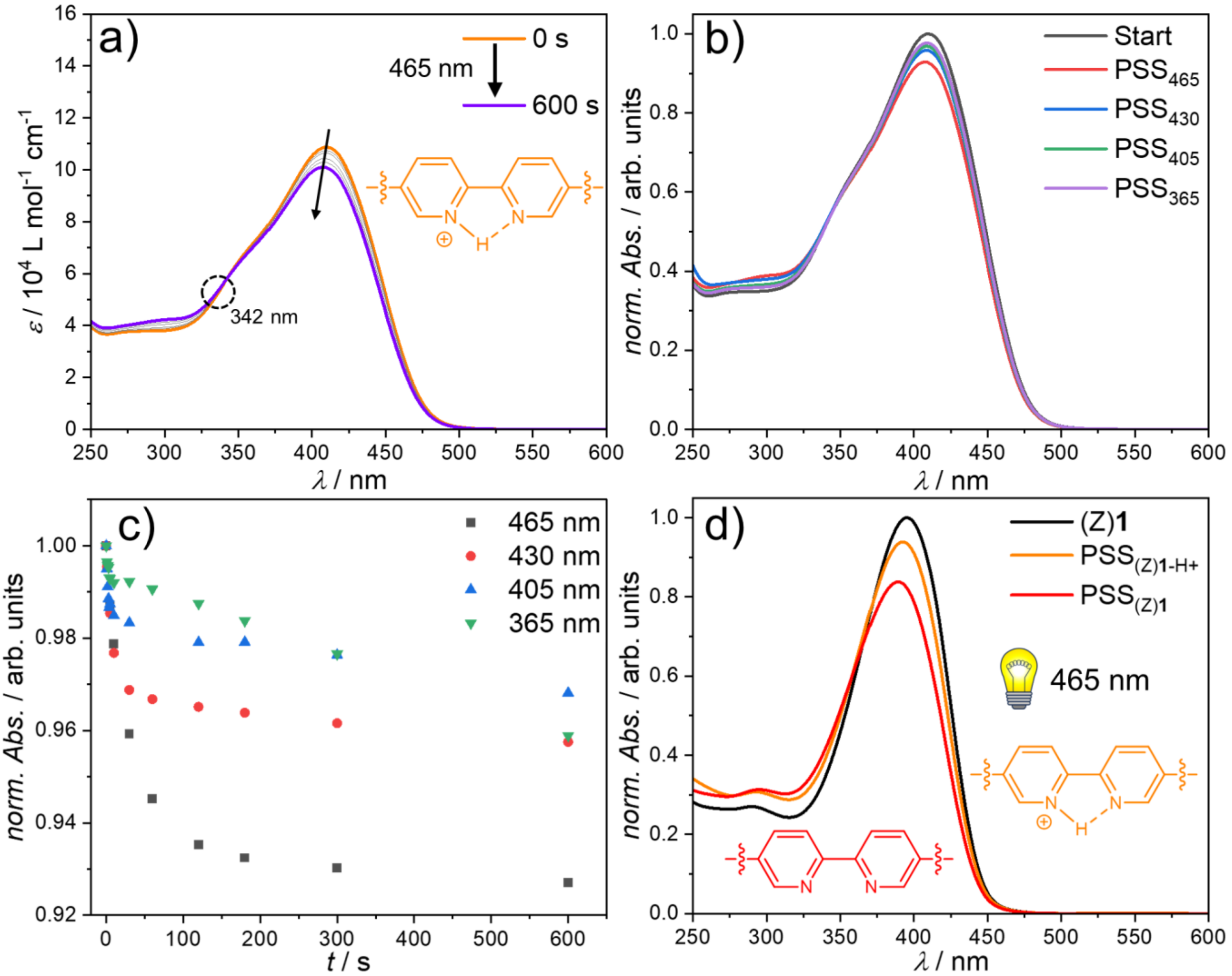

Supplementary Figure 30: Light-responsive behavior of (Z)**1**-H$^+$. a) UV-Vis spectra of (Z)**1**-H$^+$ (1×10^{-5} M, $CHCl_3$, 298 K) under irradiation with λ_{LED} = 465 nm. b) Normalized UV-Vis spectra of (Z)**1**-H$^+$ (1×10^{-5} M, $CHCl_3$, 298 K) and the resulting PSS of the irradiations with different wavelengths. c) Plot of the normalized absorbance at λ_{max} = 410 nm as a function of time for the photo-responsive reactions with different wavelengths. d) Comparison of the initial spectrum and the PSS spectra of the irradiations with different wavelengths. Spectra of the irradiations of (Z)**1**-H$^+$ and (Z)**1** with λ_{LED} = 465 nm.

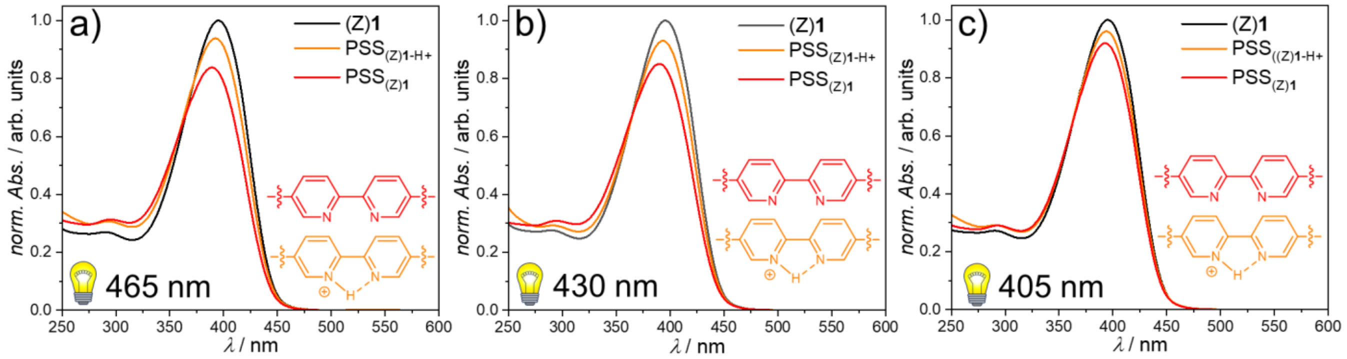

Supplementary Figure 31: Wavelength-dependent studies. Comparison of the PSS absorption spectra of (Z)**1** (red) and (Z)**1**-H$^+$ (orange) (1×10^{-5} M, $CHCl_3$, 298 K) using a) λ_{LED} = 465 nm, b) λ_{LED} = 430 nm, and c) λ_{LED} = 405 nm.

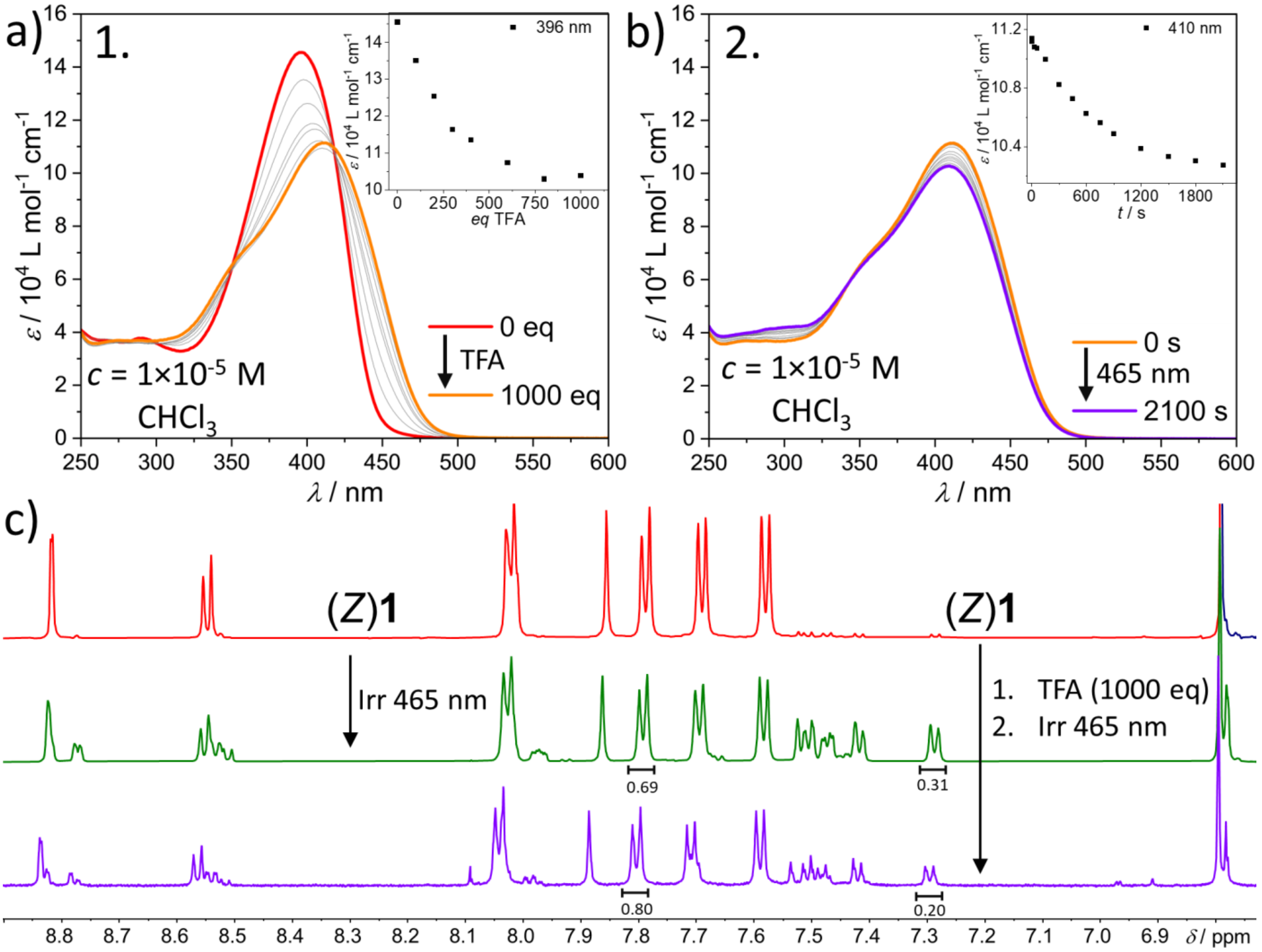

Supplementary Figure 32**:** Large-scale irradiation of (Z)**1**-H$^+$. a) UV-Vis spectra of (Z)**1** (1×10^{-5} M, $CHCl_3$, 298 K) under successive addition of TFA. Inset: plot of ε at λ = 396 nm versus the added equivalents of TFA. b) UV-Vis spectra of (Z)**1**-H$^+$ (1×10^{-5} M, $CHCl_3$, 298 K) under irradiation with λ_{LED} = 465 nm, Insets: Plot of ε at λ_{max} = 410 nm versus irradiation time. c) Sections from the aromatic region of the ^{1}H-NMR spectra of (Z)**1** (THF-d_8, 600 MHz, 299 K) before irradiation (red spectrum), after irradiation with λ_{LED} = 465 nm ($CHCl_3$, 1×10^{-4} M, 298 K, 50 min, green spectrum), and after irradiation of (Z)**1**-H$^+$ with λ_{LED} = 465 nm ($CHCl_3$, 1×10^{-5} M, 35 min, 298 K, 20 h, purple spectrum).

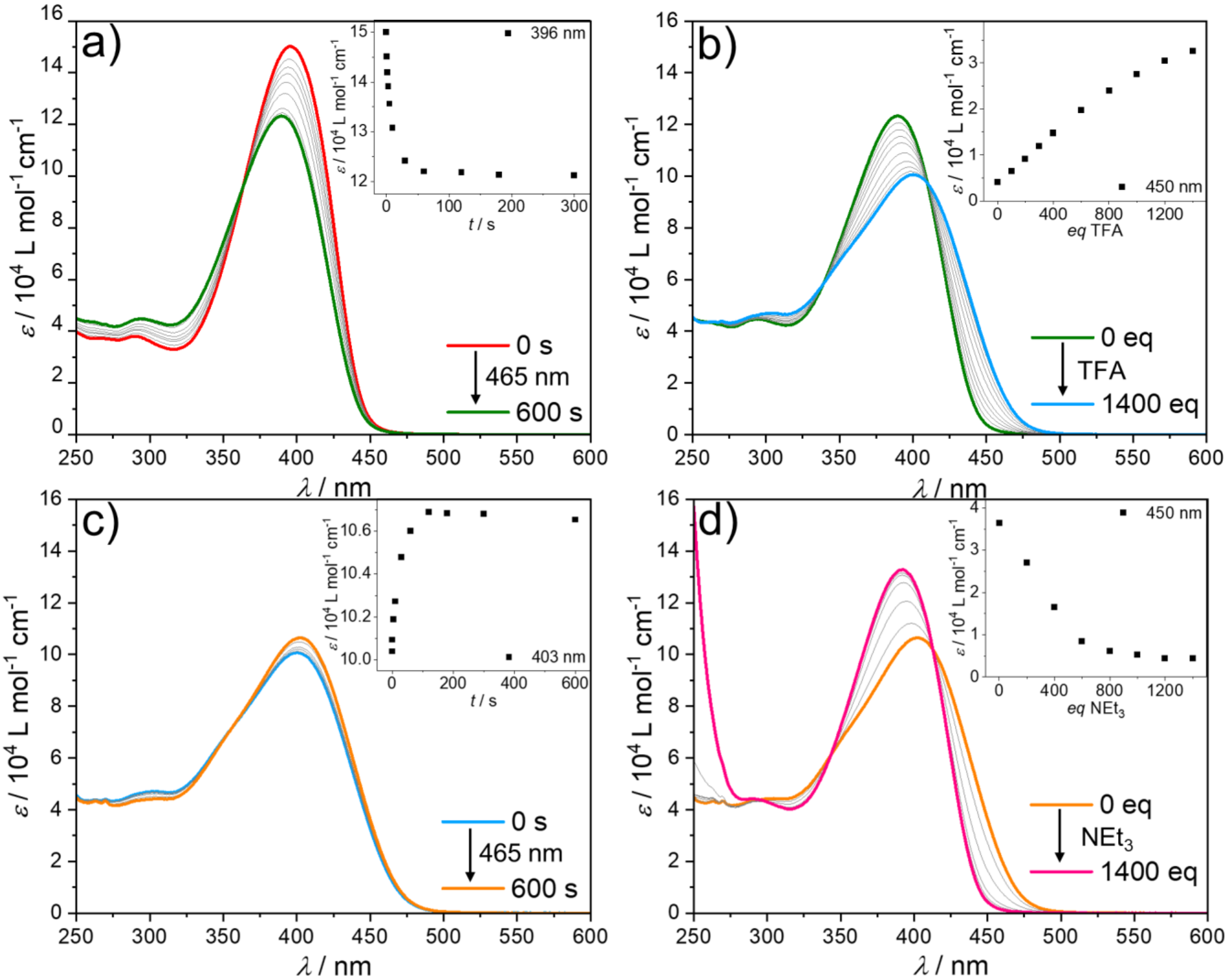

Supplementary Figure 33: Full stimuli-responsive cycle of (Z)**1**. a) UV-Vis spectra of (Z)**1** (1×10^{-5} M, $CHCl_3$, 298 K) under irradiation with λ_{LED} = 465 nm. Inset: Evolution of the absorbance at λ_{max} over the irradiation time. b) UV-Vis spectra of (E)**1** (1×10^{-5} M, $CHCl_3$, 298 K) under successive addition of TFA. Inset: course of the extinction coefficients at λ = 450 nm versus the added equivalents of TFA. c) UV-Vis spectra of (E)**1**-H^+ (1×10^{-5} M, $CHCl_3$, 298 K) under irradiation with λ_{LED} = 465 nm. Inset: Evolution of the absorbance at λ_{max} over the irradiation time. d) UV-Vis spectra of (Z)**1**-H^+ (1×10^{-5} M, $CHCl_3$, 298 K) over successive additions of NEt_3. Inset: plot of extinction coefficients at λ = 450 nm vs. equivalents of NEt_3 added.

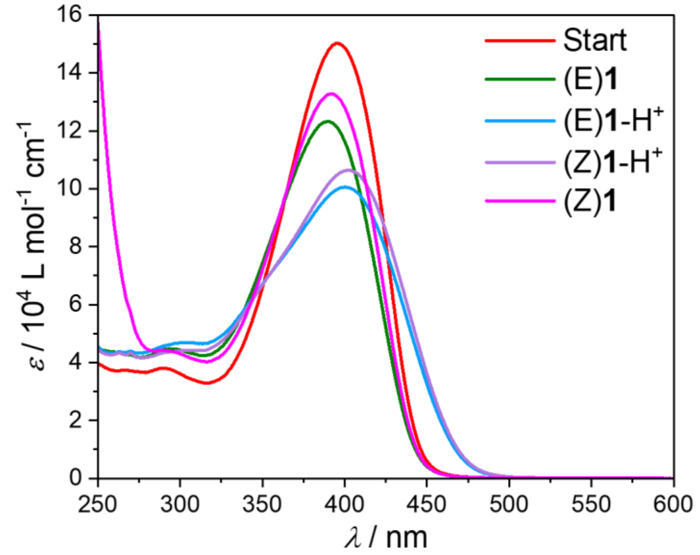

Supplementary Figure 34: Spectroscopic signatures of all species. UV-Vis spectra (1×10^{-5} M, $CHCl_3$, 298 K) of all different stages upon exposure to alternately applied stimuli.

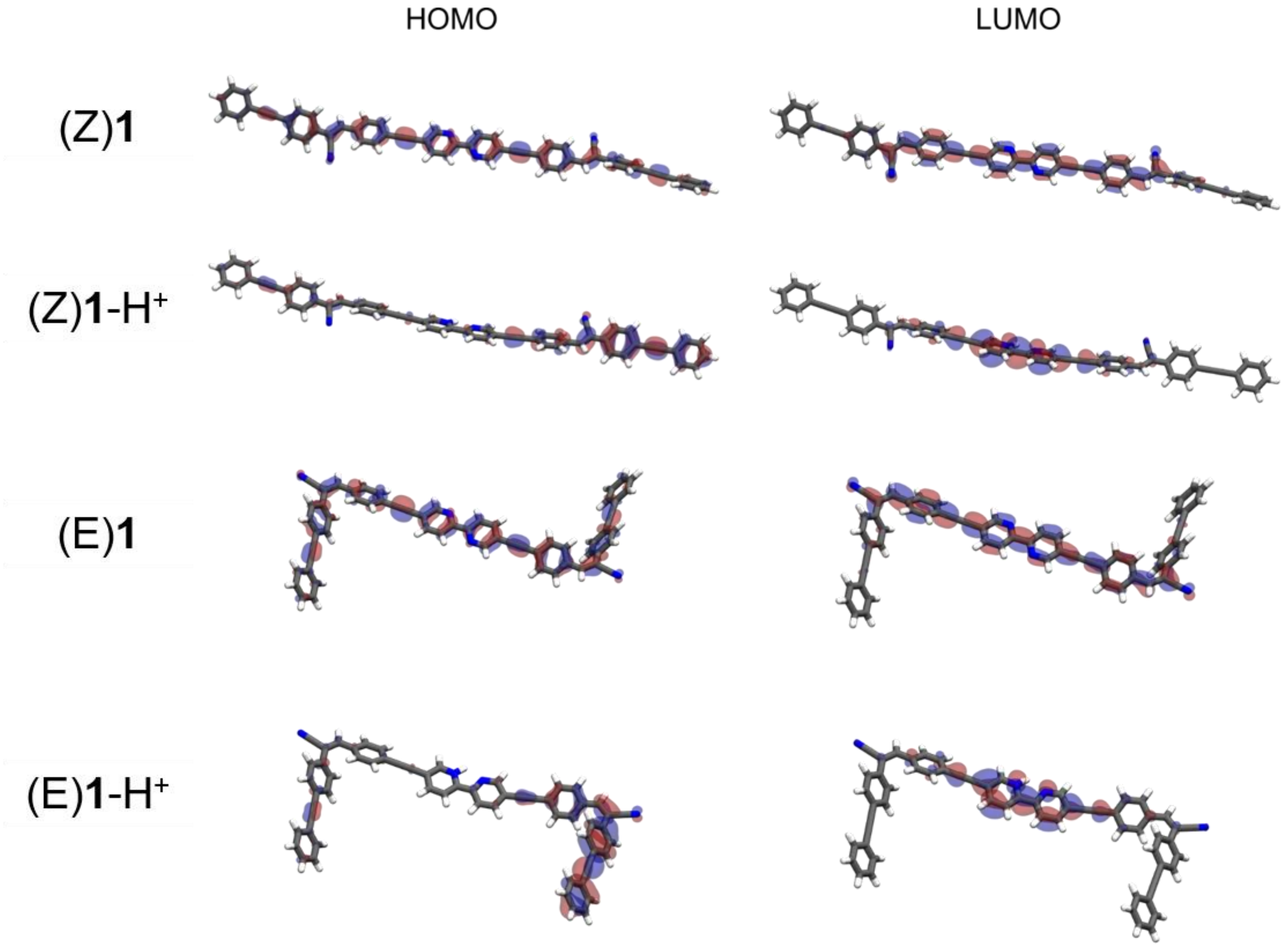

Supplementary Figure 35: HOMO/LUMO of all species. Isosurface plots of the highest occupied and lowest unoccupied molecular orbitals (HOMO/LUMO) of (Z)**1**, (Z)**1**-H^+, (E)**1**, and (E)**1**-H^+ [CAM-B3LYP/def2-TZVP/CPCM(CH_2Cl_2)].

Supplementary Table 2: Calculated excitation energies E_{ex} and oscillator strengths f for the energetically lowest singlet-singlet transitions of (Z)**1**, (Z)**1**-H^+, (E)**1**, and (E)**1**-H^+ [CAM-B3LYP/def2-TZVP/CPCM(CH_2Cl_2)]. All excitations listed are of HOMO-LUMO type.

	E_{ex} / eV	f
(Z)**1**	3.06	5.69
(E)**1**	3.21	3.32
(Z)**1**-H^+	2.77	4.89
(E)**1**-H^+	2.88	2.86

3.3 Self-Assembly in aliphatic Solvents

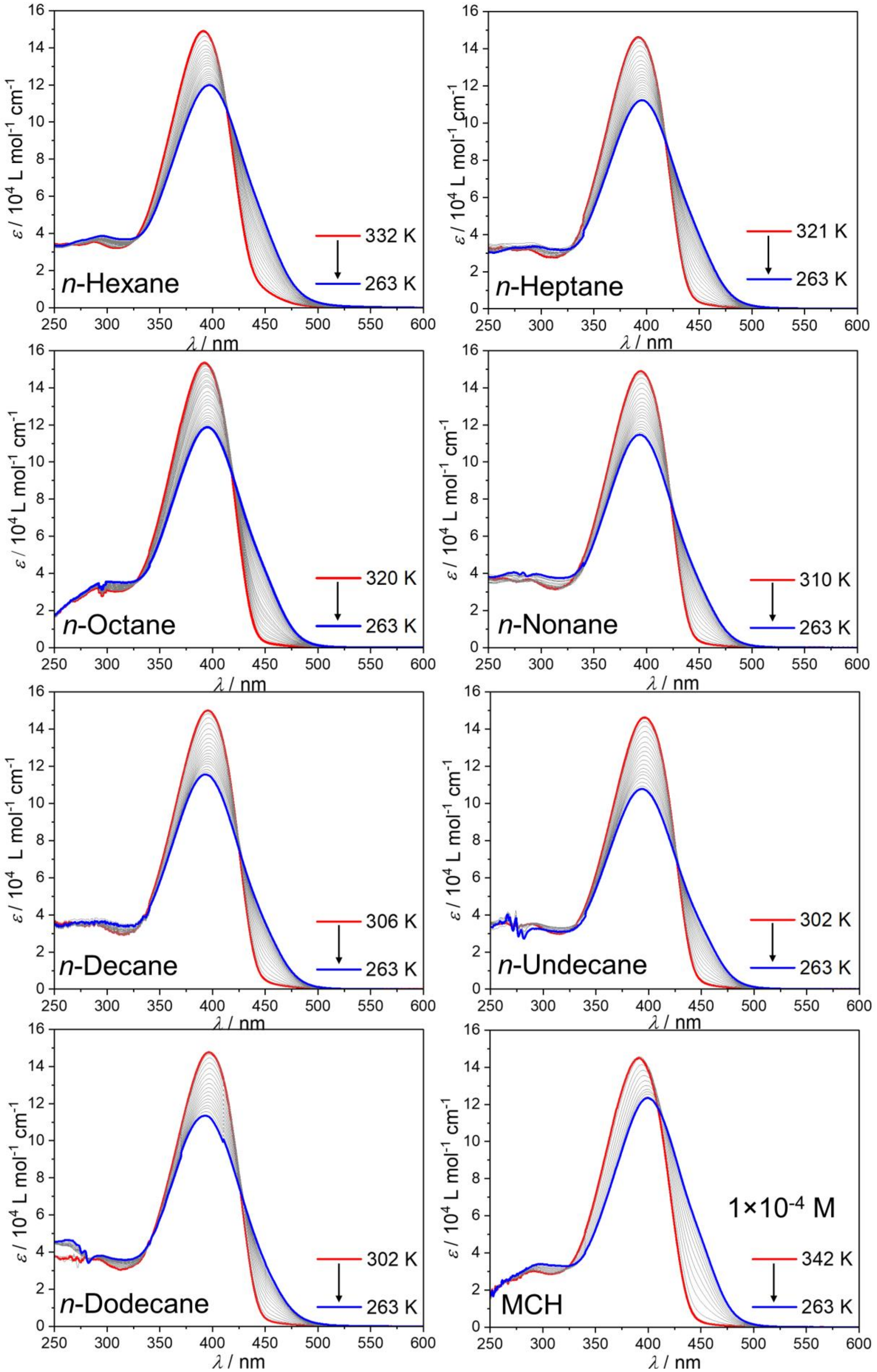

Supplementary Figure 36: Aggregation of (Z)**1** in different alkanes. VT UV-Vis spectra of (Z)**1** in different linear alkanes (1×10^{-5} M, 1 K/min) and the cyclic MCH (1×10^{-4} M, 1 K/min).

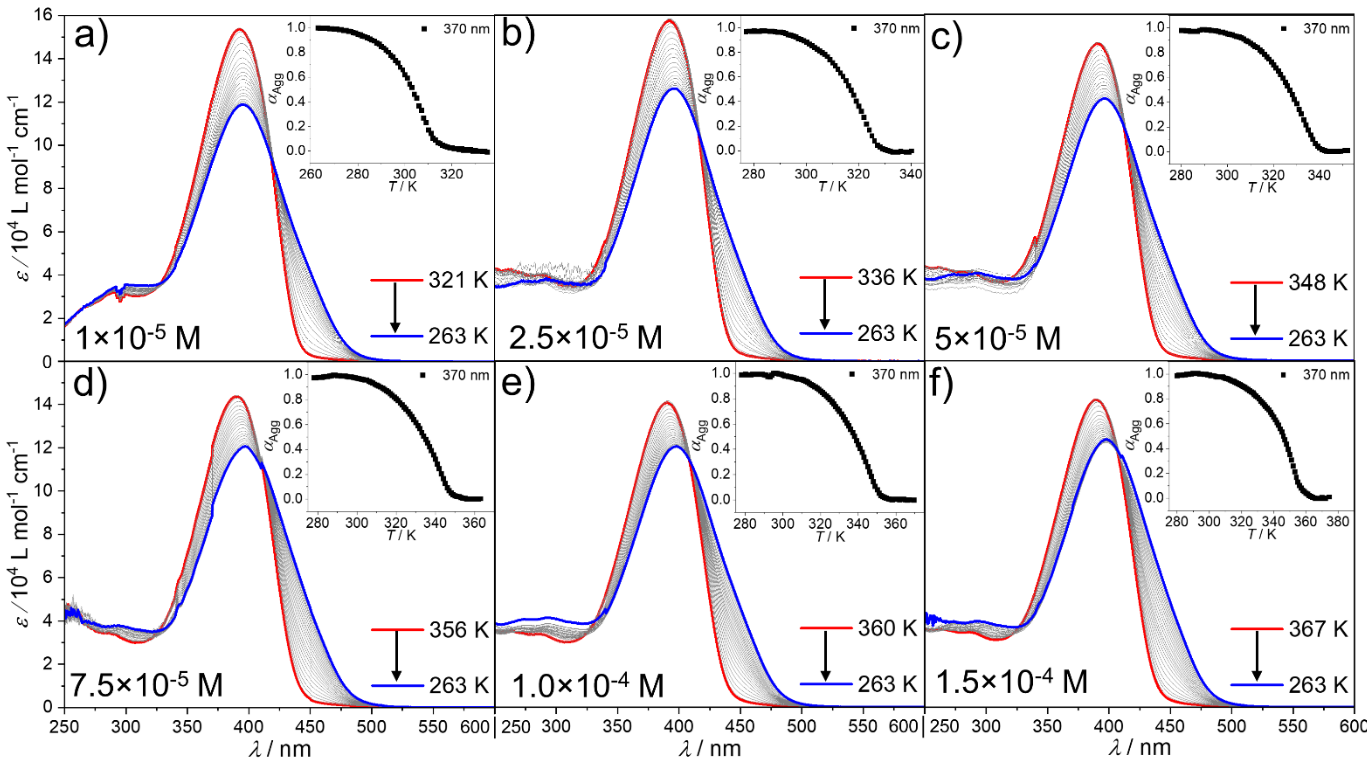

Supplementary Figure 37: Monomer-to-aggregate transformation upon cooling. VT UV-Vis spectra of (Z)**1** (*n*-octane, 1 K/min) at different concentrations [1×10^{-5} M (a), 2.5×10^{-5} M (b), 5×10^{-5} M (c), 7.5×10^{-5} M (d), 1×10^{-4} M (e), 1.5×10^{-4} M (f)]. Insets: Plot of degree of aggregation α_{Agg} vs. T.

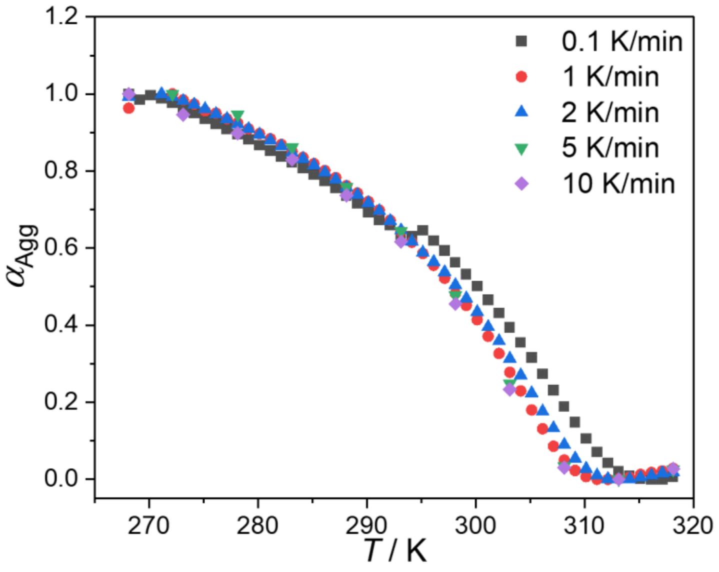

Supplementary Figure 38: Cooling-rate independent aggregation process of (Z)**1**. Plot of α_{Agg} vs. T at λ = 450 nm extracted from the temperature-dependent UV-Vis experiments (1×10^{-5} M, *n*-octane) with different cooling rates.

Supplementary Table 3: Thermodynamic parameters from the global calculation approach of the K_2-K model of the cooperative supramolecular polymerization of (Z)**1** in *n*-octane.

Solvent	ΔH^0 / kJ mol^{-1} K^{-1}	ΔH^0_{nucl} / kJ mol^{-1} K^{-1}	ΔS^0 / kJ mol^{-1} K^{-1}	ΔG^{298} / kJ mol^{-1}	σ
n-octane	-57.20 ±0.26	-21.87 ±0.82	-0.087 ±0.001	-31.33	2.26×10^{-4}

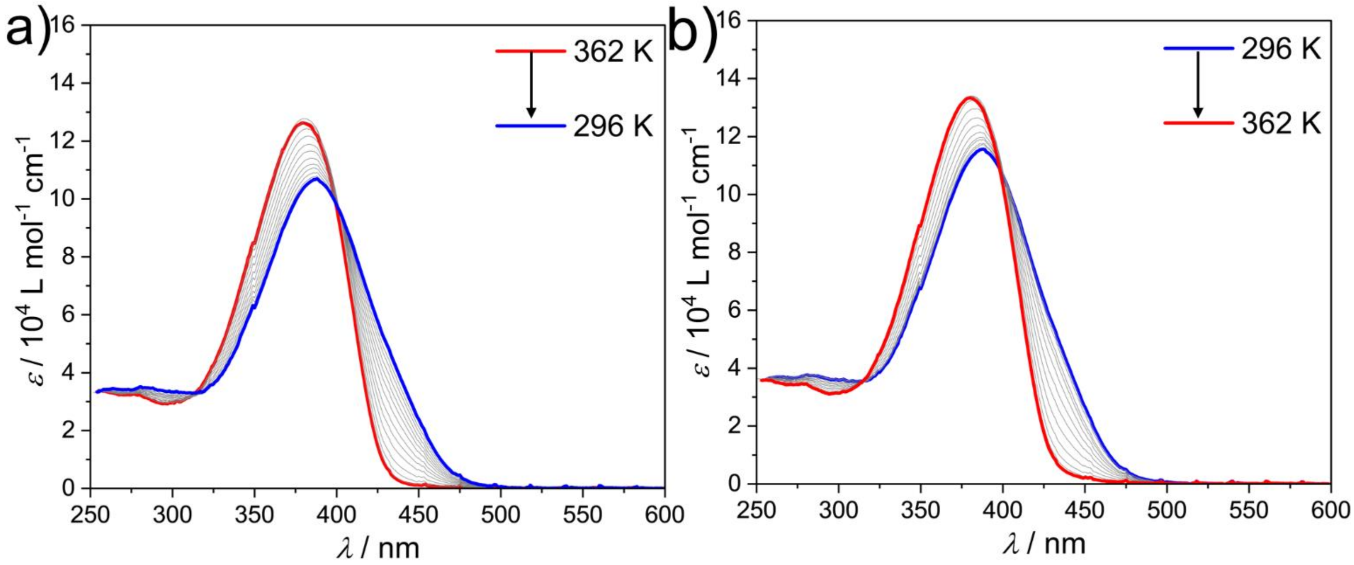

Supplementary Figure 39: Cooling-heating cycle of (Z)**1**. a) VT-UV-Vis-spectra of (Z)**1** (1×10^{-4} M, 1 K/min, *n*-octane) upon cooling. b) VT-UV-Vis-spectra of (Z)**1** (1×10^{-4} M, 1 K/min, *n*-octane) of the subsequent heating experiment.

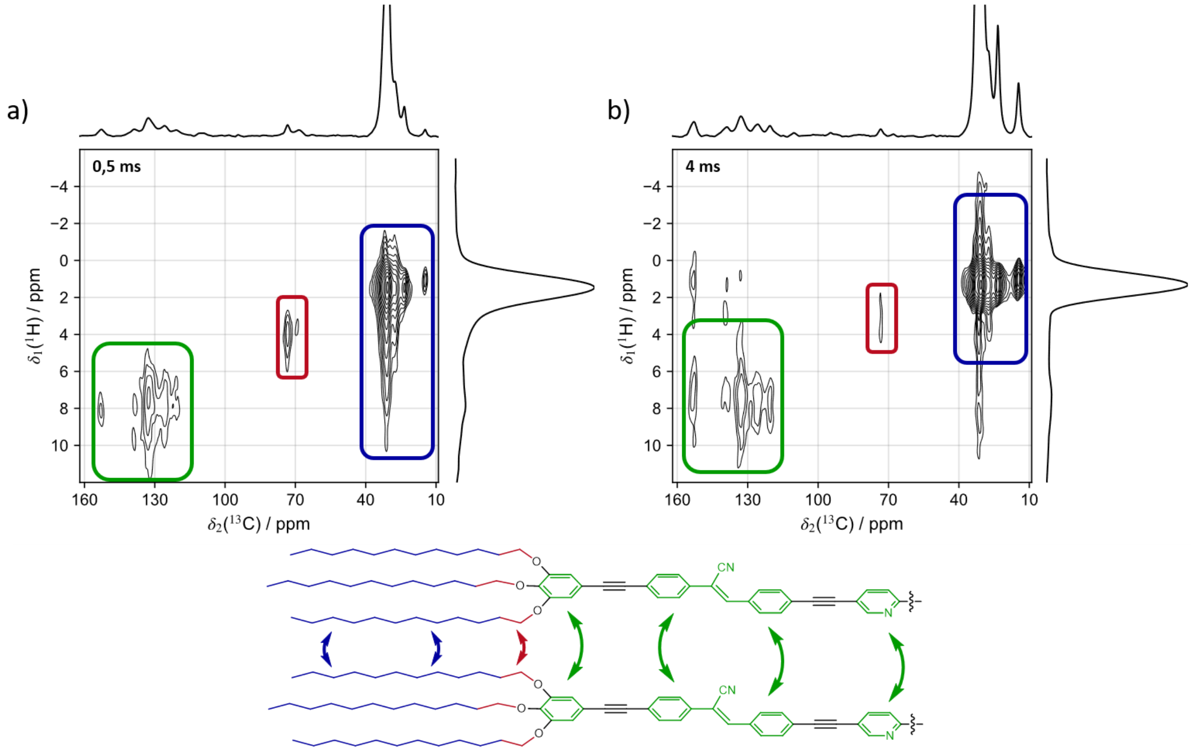

Supplementary Figure 40: Molecular packing of (Z)$\mathbf{1}_{\mathrm{Agg}}$. 2D ^{13}C{^{1}H} HETCOR NMR spectra of (Z)$\mathbf{1}_{\mathrm{Agg}}$, recorded at a magnetic field strength of 11.7 T. a) Utilizing a short CP contact time of τ_{CP} = 0.5 ms showing intramolecular and b) using a long CP contact time of τ_{CP} = 4 ms showing intermolecular ^{1}H-^{13}C correlations. The intermolecular ^{1}H-^{13}C correlations are consistent with the schematic representation of a dimer (in color).

The sample for the solid-state NMR experiments was prepared by dissolving a large amount of (Z)**1** (50 mg) in hot *n*-octane (20 mL, 1.3×10^{-3} M) followed by slow cooling to r.t.. Subsequently, the solvent was removed by slow evaporation prior to the measurements. Signal assignment was carried out *via* standard solution state NMR experiments before transferring the aggregate to the solid state and additionally confirmed utilizing 2D ^{13}C{^{1}H} HETCOR NMR experiments with a short CP contact time of $\tau_{\mathrm{CP}} = 0.5$ ms, showing only intramolecular correlations (Supplementary Figure 40a).

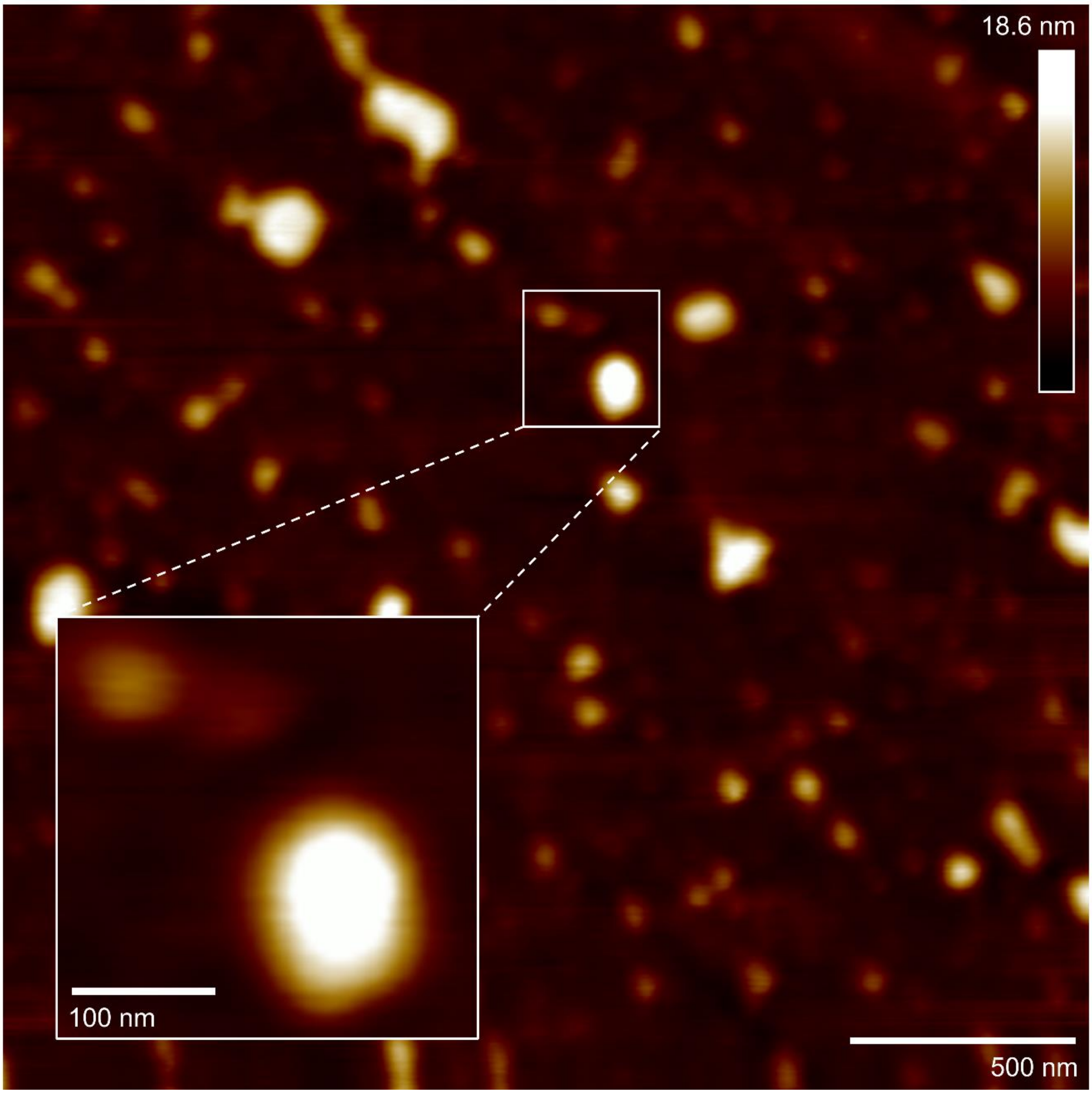

Supplementary Figure 41: Aggregate morphology of (Z)**1**$_{Agg}$. AFM image of (Z)**1**$_{Agg}$ after spin-coating a 10 µM solution in *n*-octane onto an HOPG surface (for detailed sample preparation protocols see section 1.1).

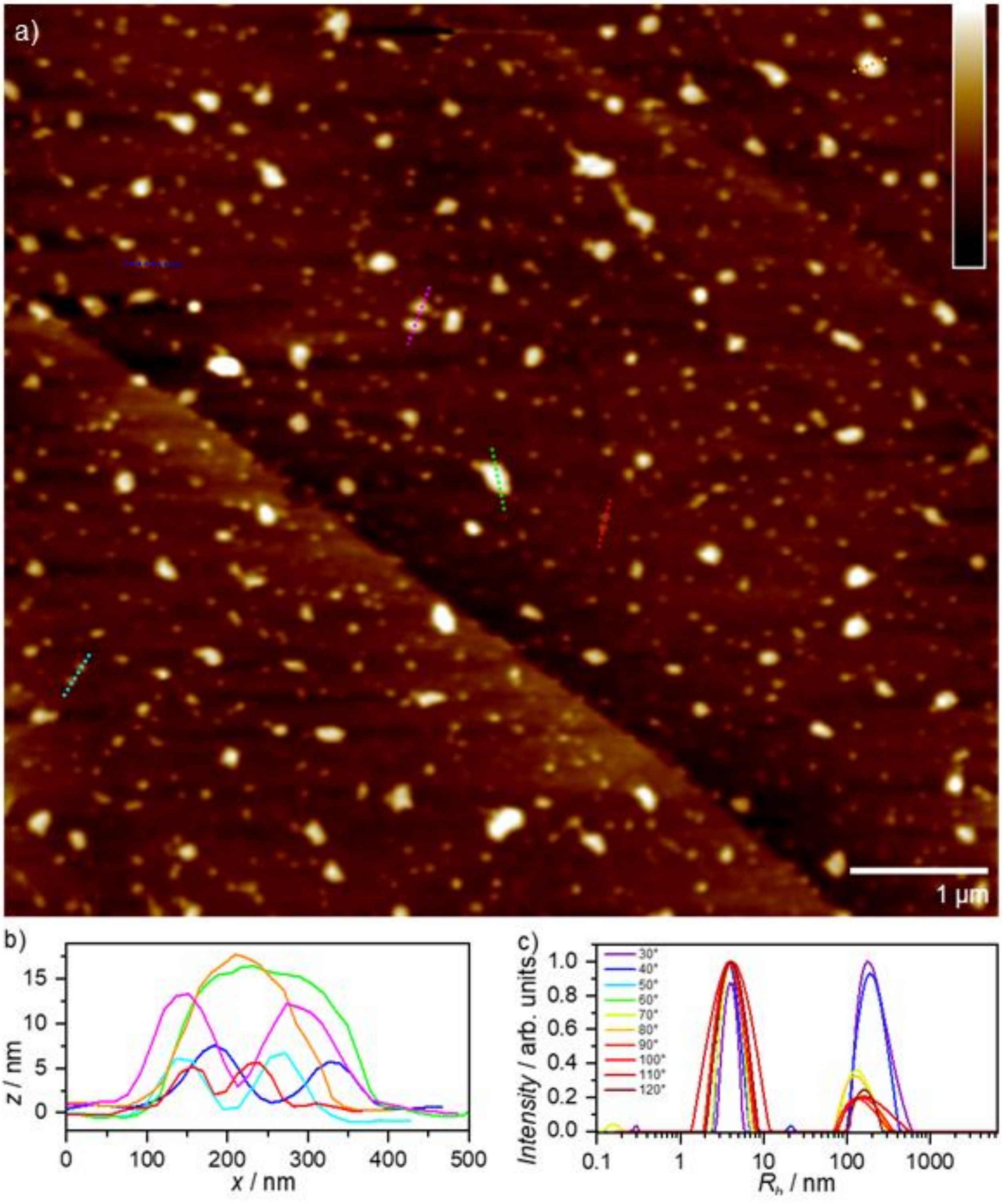

Supplementary Figure 42: Morphology and size distribution analysis of (Z)$\mathbf{1}_{Agg}$. a) AFM image of (Z)$\mathbf{1}_{Agg}$ after spin-coating a 10 µM solution in *n*-octane onto an HOPG surface (for detailed sample preparation protocols see section 1.1). b) height profiles of selected aggregates in a). c) corresponding size distributions derived from DLS measurements of a 100 µM solution in *n*-octane.

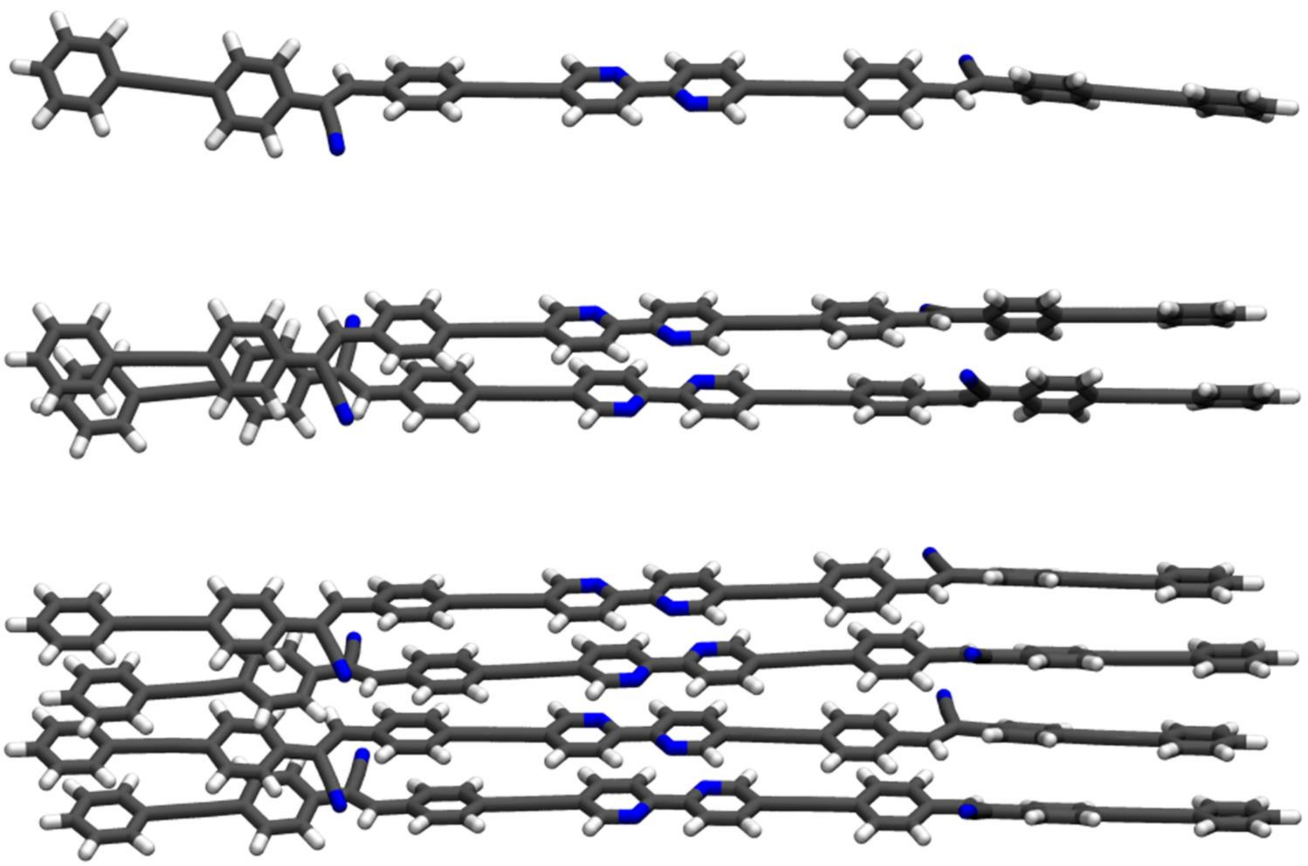

Supplementary Figure 43: Computed molecular packing of (Z)**1**. Geometry-optimized [level of theory: GFN2-xTB (6.4.1)] molecular structures of (Z)**1**$_{Agg}$ as monomer (top), dimer (middle) and tetramer (bottom) (GFN2-xTB (6.4.1)). The peripheral dodecyl chains were removed to simplify the calculations.

3.4 Supramolecular adaptive behavior

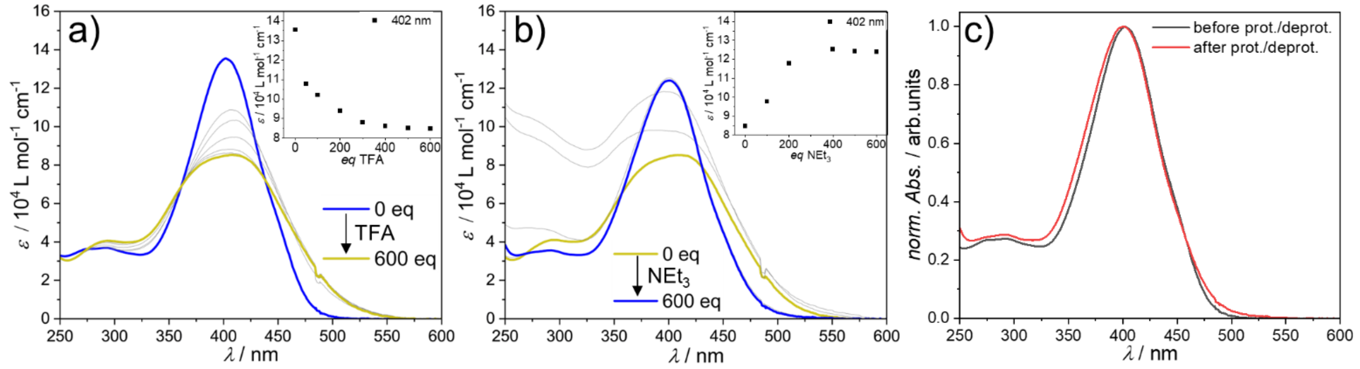

Supplementary Figure 44: Protonation-deprotonation of (Z)**1** in *n*-octane. a) UV-Vis spectra of (Z)**1**$_{Agg}$ (1×10^{-5} M, *n*-octane, 298 K) under successive addition of TFA. Inset: Plot of the extinction coefficient ε at λ_{max} *vs*. the amount of added TFA. b) UV-Vis spectra of (Z)**1**-H^{+}$_{Agg}$ (1×10^{-5} M, *n*-octane, 298 K, + 600 eq. TFA) under successive addition of NEt_3. Inset: plot of the extinction coefficient ε at λ_{max} vs. the amount of added NEt_3. c) Comparison of the normalized UV-Vis spectra of (Z)**1**$_{Agg}$ (1×10^{-5} M, *n*-octane, 298 K) before (gray) and after a cycle of protonation/deprotonation with TFA/NEt_3 (red).

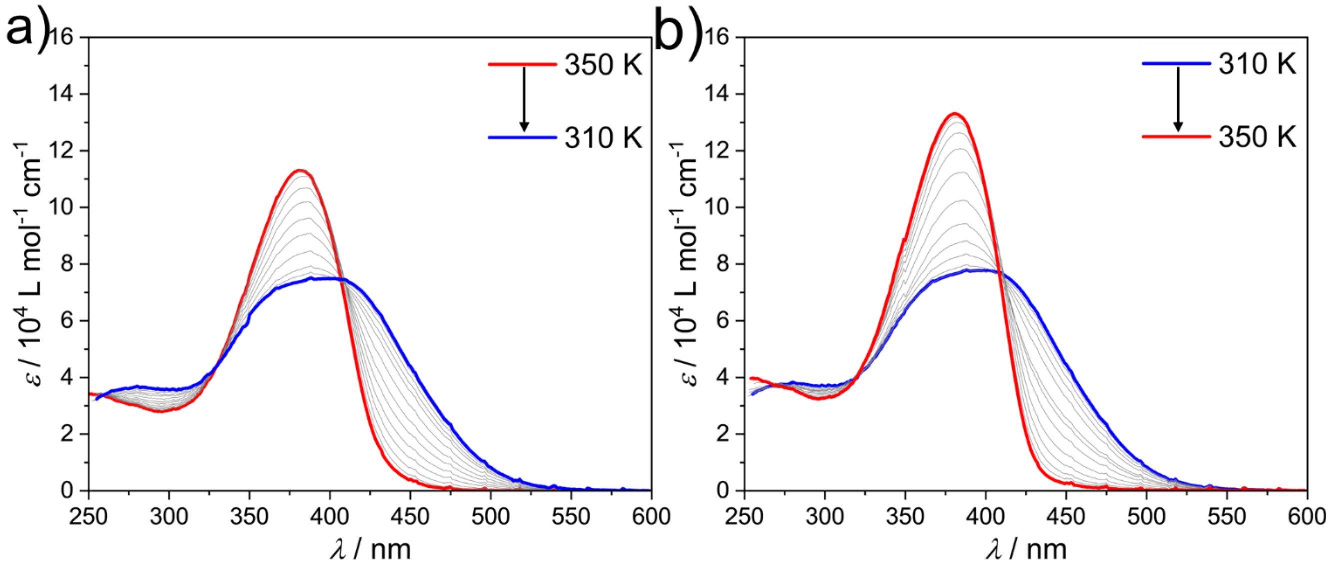

Supplementary Figure 45: Cooling-heating cycle of (Z)**1**-H$^+$. a) VT-UV-Vis spectra of (Z)**1**-H$^+$ (1×10^{-5} M, 1 K/min, *n*-octane) upon cooling. b) VT-UV-Vis spectra of (Z)**1**-H$^+$ (1×10^{-5} M, 1 K/min, *n*-octane) of the subsequent heating experiment.

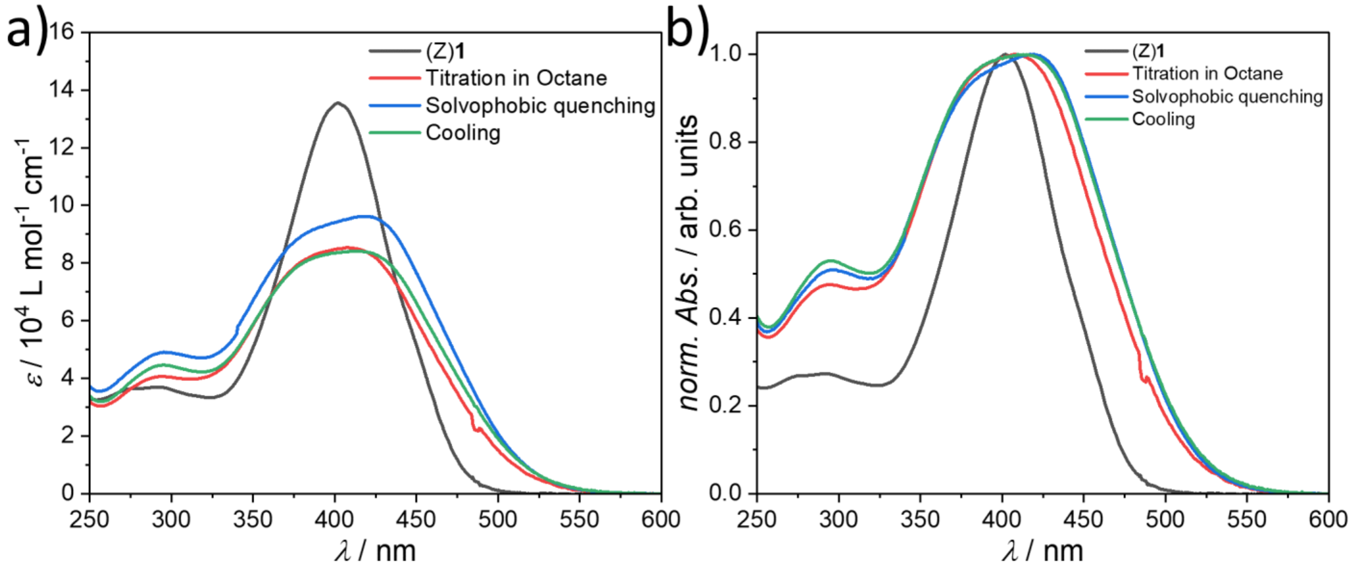

Supplementary Figure 46: Comparison of different aggregation-inducing approaches of (Z)**1**-H$^+$. a) UV-Vis spectra of (Z)**1**-H^+_{Agg} (1×10^{-5} M, *n*-octane, 298 K) obtained by various techniques (titration, solvophobic quenching, cooling). b) Normalized UV-Vis spectra of (Z)**1**-H^+_{Agg} (1×10^{-5} M, *n*-octane, 298 K) obtained by various techniques (titration, solvophobic quenching, cooling).

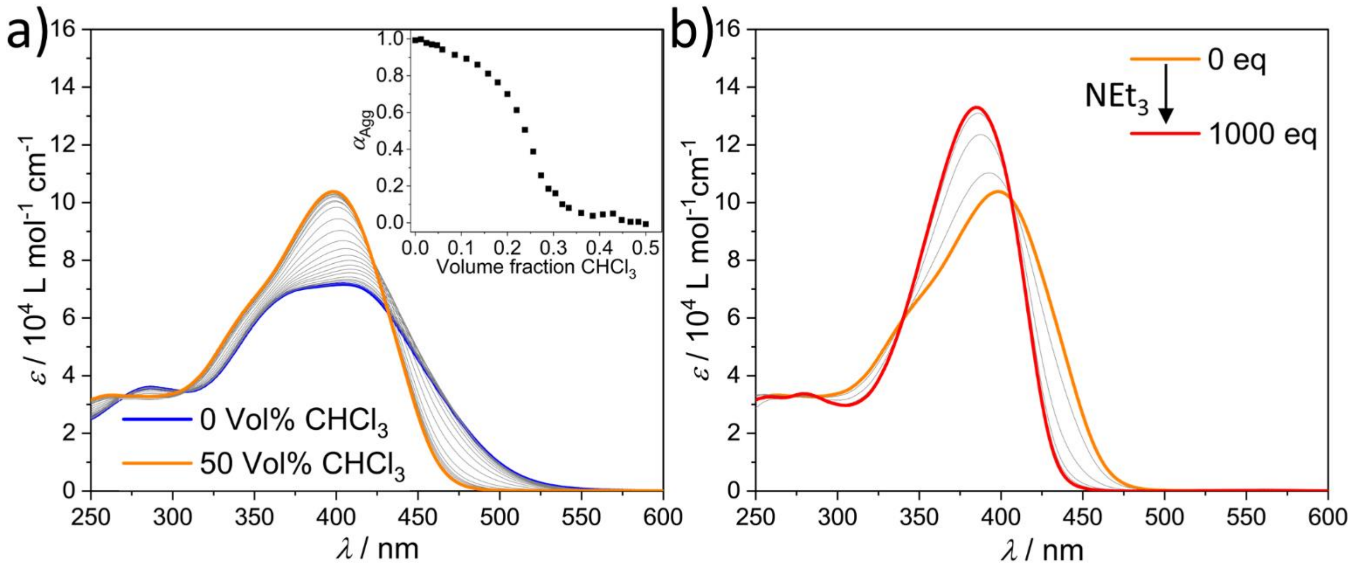

Supplementary Figure 47: Denaturation of (Z)**1**-H^+_{Agg}. a) UV-Vis-spectra of the denaturation experiment of (Z)**1**-H^+_{Agg} (1×10^{-5} M, *n*-octane, 298 K). The experiment was conducted by the addition of incremental amounts of an equimolar monomer solution of (Z)**1**-H^+ in $CHCl_3$ to the aggregate solution to achieve concentration consistency. Inset: Plot of α_{Agg} vs. volume fraction of $CHCl_3$. b) UV-Vis spectra of the consecutive deprotonation with NEt_3.

Supplementary Table 4: Thermodynamic parameters derived from UV-Vis cooling experiments of (Z)**1**-H^+ at different concentrations (*n*-octane, 1 K/min).

c / M	ΔH^0 / kJ mol^{-1} K^{-1}	ΔH^0_{nucl} / kJ mol^{-1} K^{-1}	ΔS^0 / kJ mol^{-1} K^{-1}	T_e / K	ΔG^{298} / kJ mol^{-1}	σ
2.5×10^{-6}	-145.41 ±13.07	-3.62 ±1.28	-0.335 ±0.041	328.87 ±1.08	-45.56	2.66×10^{-1}
5.0×10^{-6}	-177.09 ±21.30	-3.23 ±1.74	-0.418 ±0.064	341.22 ±1.28	-52.61	3.19×10^{-1}
7.5×10^{-6}	-171.64 ±6.27	-6.43 ±0.51	-0.393 ±0.018	349.81 ±0.30	-54.60	1.10×10^{-1}
1.0×10^{-5}	-164.11 ±4.89	-6.79 ±0.44	-0.366 ±0.0	355.63 ±0.27	-55.06	1.01×10^{-1}
1.5×10^{-5}	-166.90 ±5.47	-6.33 ±0.48	-0.377 ±0.016	355.44 ±0.30	-54.44	1.18×10^{-1}
mean	-165.03 ±11.18	-5.28 ±0.89	-0.378 ±0.028	-	-52.45	1.8×10^{-1}
[a]1.0×10^{-5} ((Z)**1**)	-61.45 ±0.50	-13.72 ±0.19	-0.103 ±0.002	308.99 ±0.09	-30.69	4.79×10^{-3}

[a]For a better comparison, the thermodynamic parameters were obtained by a single fit according to the K_2-K model at this concentration.

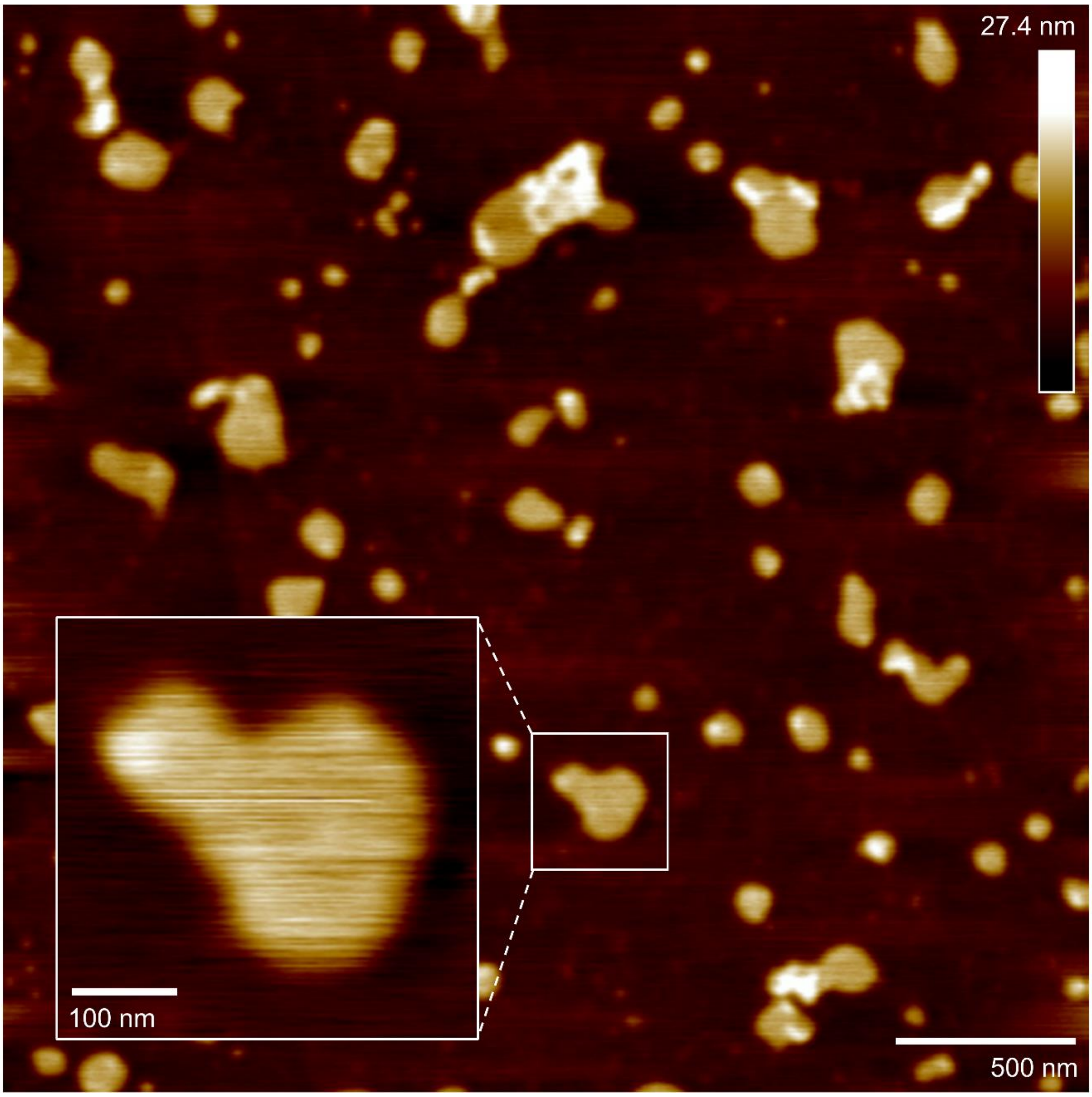

Supplementary Figure 48: Aggregate morphology of (Z)**1**-H^{+}_{Agg}. AFM image of (Z)**1**-H^{+}_{Agg} after spin-coating a 10 µM solution in *n*-octane onto an HOPG surface (for detailed sample preparation protocols see section 1.1).

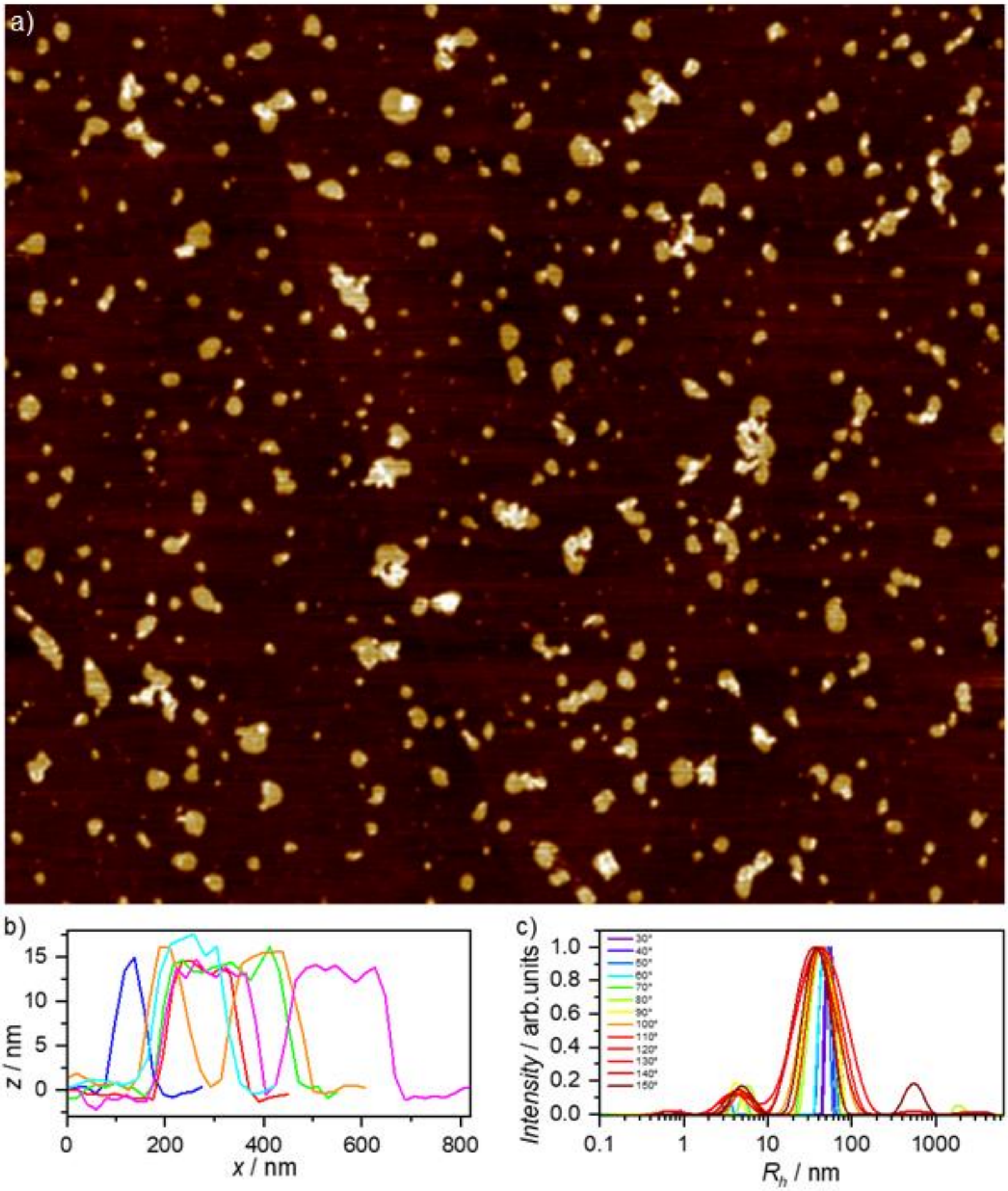

Supplementary Figure 49: Morphology and size distribution analysis of (Z)**1**-H^{+}_{Agg}. a) AFM image of (Z)**1**-H^{+}_{Agg} after spin-coating a 10 µM solution in *n*-octane onto an HOPG surface (for detailed sample preparation protocols see section 1.1). b) height profiles of selected aggregates in a). c) corresponding size distributions derived from DLS measurements of a 100 µM solution in *n*-octane.

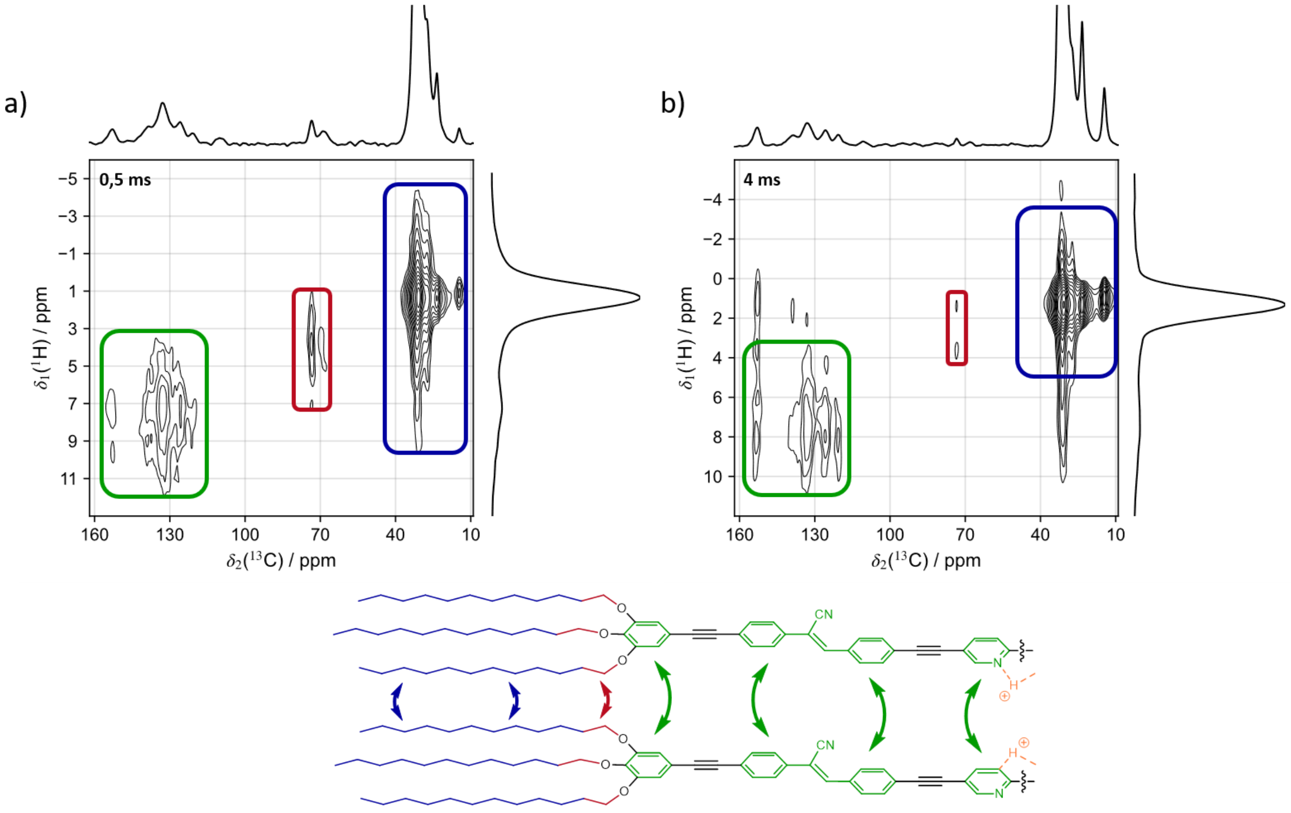

Supplementary Figure 50: Molecular packing of (Z)**1**-H^+_{Agg}. a) 2D $^{13}C\{^1H\}$ HETCOR NMR spectra of (Z)**1**-H^+_{Agg}, recorded at a magnetic field strength of 11.7 T. a) Utilizing a short CP contact time of τ_{CP} = 0.5 ms showing intramolecular and b) using a long CP contact time of τ_{CP} = 4 ms showing intermolecular ^1H-^{13}C correlations. The intermolecular ^1H-^{13}C correlations are consistent with the schematic representation of a dimer (in color).

The sample for the solid-state NMR experiments was prepared by adding 1000 eq of TFA to a concentrated solution of (Z)$\mathbf{1}_{Agg}$ in octane (55 mg in 20 mL) under slow stirring for 2 days. The solvent was removed by slow evaporation and the aggregate was obtained as an intense red solid. The signal assignment was done using standard solution state NMR experiments and confirmed utilizing $^{13}C\{^1H\}$ HETCOR NMR experiments with a short CP contact time of τ_{CP} = 0.5 ms showing only intramolecular correlations in the solid state (Supplementary Figure 50a). To gain information about the supramolecular organization, a CP contact time of τ_{CP} = 4 ms was utilized, exhibiting not only intra- but also intermolecular ^1H-^{13}C correlations.

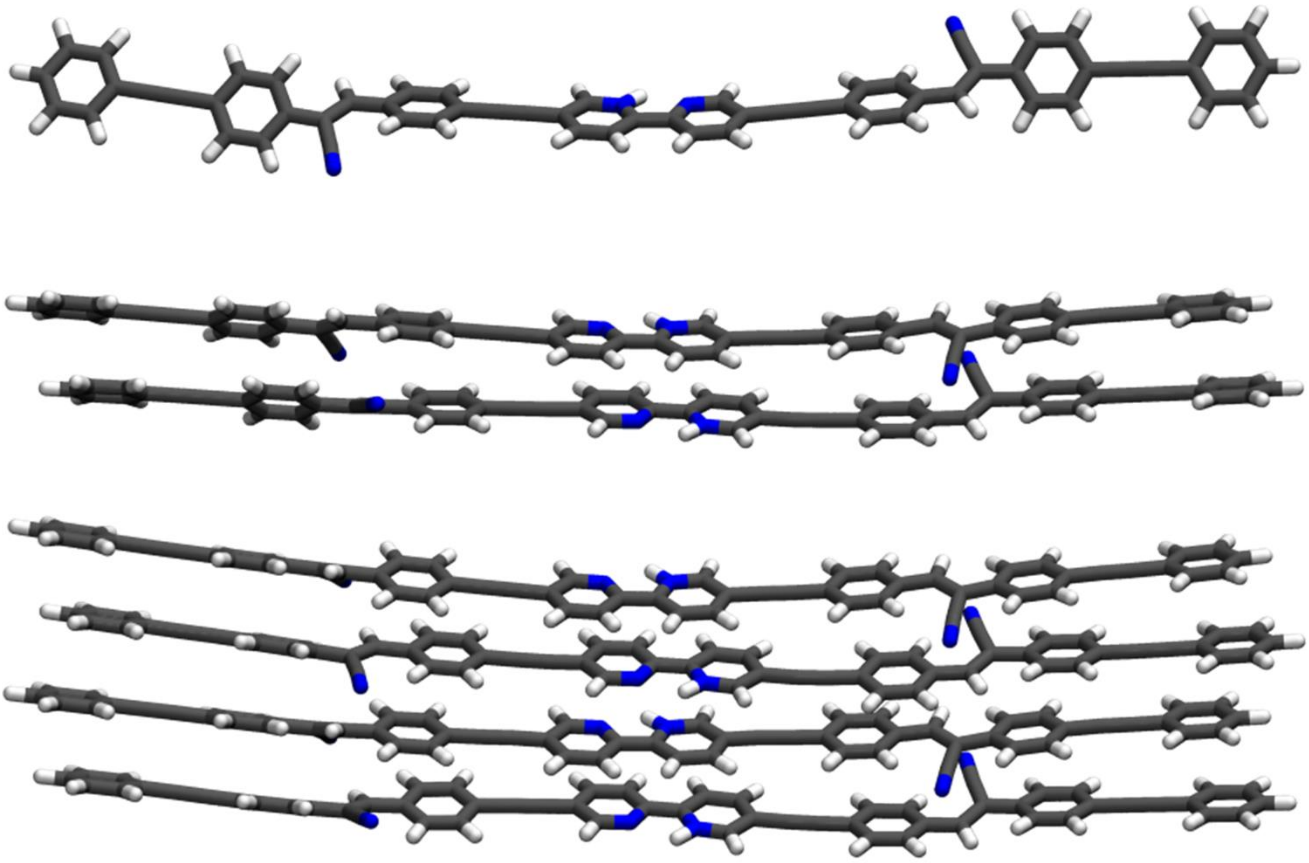

Supplementary Figure 51: Computed molecular packing of (Z)**1**-H$^+$. Geometry-optimized [level of theory: GFN2-xTB (6.4.1)] molecular structures of (Z)**1**-H$^+$ as monomer (top), dimer (middle) and tetramer (bottom) (GFN2-xTB (6.4.1)). The peripheral dodecyl chains were removed to simplify the calculations.

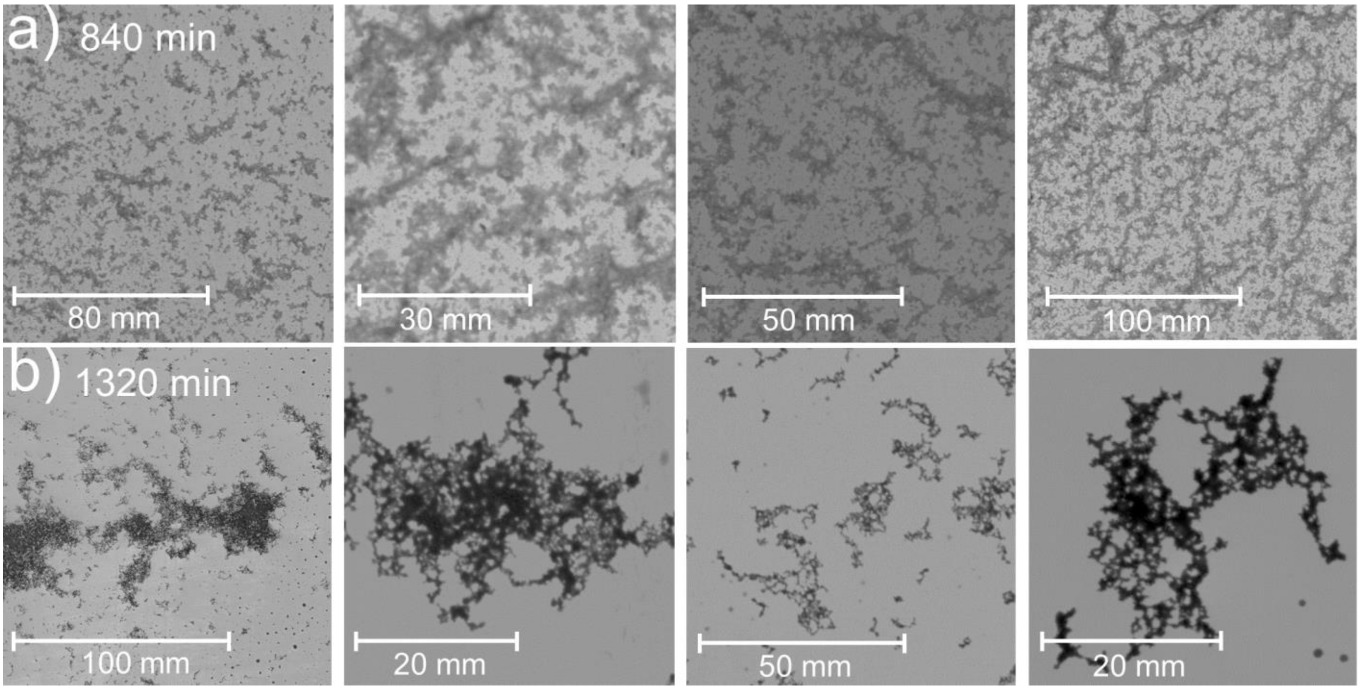

Supplementary Figure 52: Aggregate morphology of (Z)$\mathbf{1}_{Photo.}$ SEM images of (Z)$\mathbf{1}_{Photo}$ (1×10^{-4} M, *n*-octane, 298 K) with λ_{LED} = 465 nm after a) 840 min and b) 1320 min.

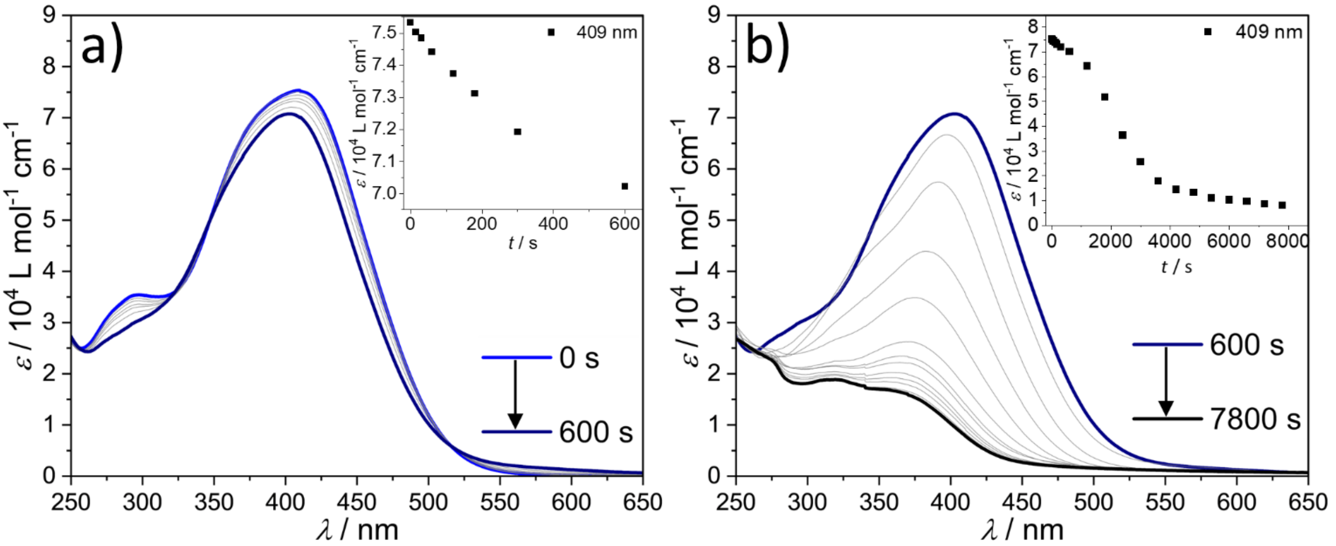

Supplementary Figure 53: Light-responsive behavior of (Z)**1**-H$^+_{Agg}$. a) UV-Vis spectra of (Z)**1**-H$^+_{Agg}$ (1×10^{-5} M, *n*-octane, 298 K) under irradiation with λ_{LED} = 465 nm up to an irradiation time of 10 min. Inset: Plot of extinction coefficient ε at λ_{max} = 409 nm vs. irradiation time. b) UV-Vis spectra of (Z)**1**-H$^+_{Agg}$ (1×10^{-5} M, *n*-octane, 298 K) under the irradiation with λ_{LED} = 465 nm at long exposure time. Inset: Plot of the extinction coefficient ε at λ_{max} = 409 nm against the irradiation time.

As demonstrated by these experiments, irradiation of (Z)**1**-H$^+_{Agg}$ for over 600 seconds leads to decomposition.

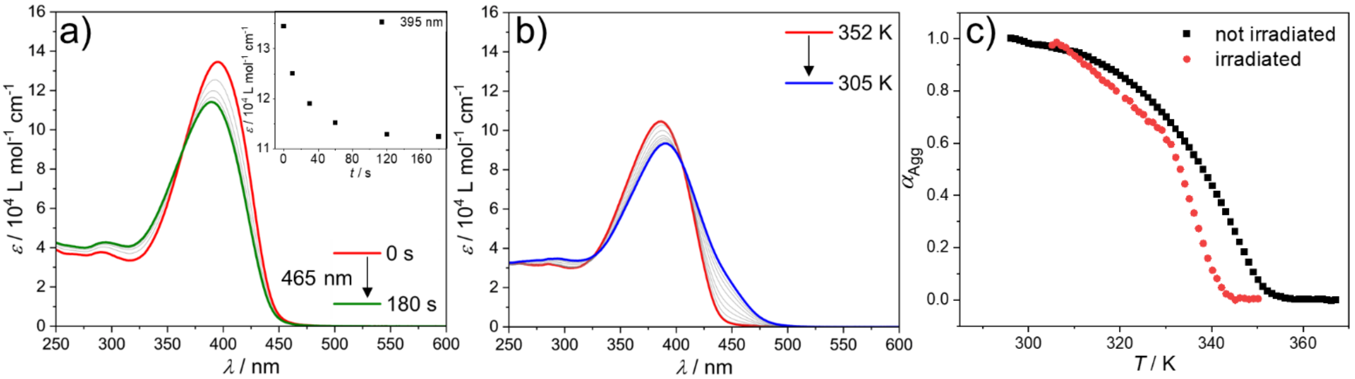

Supplementary Figure 54: Combination of irradiation and subsequent aggregation of (Z)**1**. a) UV-Vis spectra of (Z)**1** (1×10^{-5} M, $CHCl_3$, 298 K) under irradiation with λ_{LED} = 465 nm. Inset: Plot of the ε at λ_{max} *vs*. the irradiation time. b) UV-Vis cooling experiment of this 69:31 (E)**1**:(Z)**1** mixture in *n*-octane. c) comparison of the cooling curves for the aggregates of pure (Z)**1** (black plot) and the corresponding mixture 69:31 (E)**1**:(Z)**1** (red plot).

4. Supplementary references

1. Marion, D., Ikura, M., Tschudin, R. & Bax, A. Rapid recording of 2D NMR spectra without phase cycling. Application to the study of hydrogen exchange in proteins. *J. Magn. Reson.* **85**, 393–399 (1989).

2. Bannwarth, C., Ehlert, S. & Grimme, S. GFN2-xTB-An Accurate and Broadly Parametrized Self-Consistent Tight-Binding Quantum Chemical Method with Multipole Electrostatics and Density-Dependent Dispersion Contributions. *J. Chem. Theory Comput.* **15**, 1652–1671 (2019).

3. Balasubramani, S. G. *et al.* TURBOMOLE: Modular program suite for ab initio quantum-chemical and condensed-matter simulations. *J. Chem. Phys.* **152**, 184107 (2020).

4. Zhao, Y. & Truhlar, D. G. Design of density functionals that are broadly accurate for thermochemistry, thermochemical kinetics, and nonbonded interactions. *J. Phys. Chem. A* **109**, 5656–5667 (2005).

5. Weigend, F. & Ahlrichs, R. Balanced basis sets of split valence, triple zeta valence and quadruple zeta valence quality for H to Rn: Design and assessment of accuracy. *PCCP* **7**, 3297–3305 (2005).

6. Grimme, S., Antony, J., Ehrlich, S. & Krieg, H. A consistent and accurate ab initio parametrization of density functional dispersion correction (DFT-D) for the 94 elements H-Pu. *J. Chem. Phys.* **132**, 154104 (2010).

7. Grimme, S., Ehrlich, S. & Goerigk, L. Effect of the damping function in dispersion corrected density functional theory. *J. Comput. Chem.* **32**, 1456–1465 (2011).

8. Klamt, A. The COSMO and COSMO-RS solvation models. *WIREs Comput. Mol. Sci.* **1**, 699–709 (2011).

9. Klamt, A. The COSMO and COSMO-RS solvation models. *WIREs Comput. Mol. Sci.* **8**, e1338 (2018).

10. Becke, A. D. Density-functional exchange-energy approximation with correct asymptotic behavior. *Phys. Rev. A* **38**, 3098–3100 (1988).

11. Perdew, J. P. Density-functional approximation for the correlation energy of the inhomogeneous electron gas. *Phys. Rev. B* **33**, 8822–8824 (1986).

12. Yanai, T., Tew, D. P. & Handy, N. C. A new hybrid exchange–correlation functional using the Coulomb-attenuating method (CAM-B3LYP). *Chem. Phys. Lett.* **393**, 51–57 (2004).

13. Barone, V. & Cossi, M. Quantum Calculation of Molecular Energies and Energy Gradients in Solution by a Conductor Solvent Model. *J. Phys. Chem. A* **102**, 1995–2001 (1998).

14. Unsleber, J. P. *et al.* Serenity: A subsystem quantum chemistry program. *J. Comput. Chem.* **39**, 788–798 (2018).

15. Niemeyer, N. *et al.* The subsystem quantum chemistry program Serenity. *WIREs Comput. Mol. Sci.* **13**, e1647 (2023).

16. Barton, D. *et al. qcserenity/serenity: Release 1.5.2* (Zenodo, 2023).

17. Maeda, H. *et al.* Solvent-assisted organized structures based on amphiphilic anion-responsive pi-conjugated systems. *Chem. Eur. J.* **15**, 3706–3719 (2009).

18. Tanabe, K., Suzui, Y., Hasegawa, M. & Kato, T. Full-color tunable photoluminescent ionic liquid crystals based on tripodal pyridinium, pyrimidinium, and quinolinium salts. *J. Am. Chem. Soc.* **134**, 5652–5661 (2012).

19. Cardolaccia, T., Li, Y. & Schanze, K. S. Phosphorescent platinum acetylide organogelators. *J. Am. Chem. Soc.* **130**, 2535–2545 (2008).

20. Ayme, J.-F. *et al.* Pentameric circular iron(II) double helicates and a molecular pentafoil knot. *J. Am. Chem. Soc.* **134**, 9488–9497 (2012).

21. Eikelder, H. M. M. ten, Markvoort, A. J., Greef, T. F. A. de & Hilbers, P. A. J. An equilibrium model for chiral amplification in supramolecular polymers. *J. phys. Chem. B* **116**, 5291–5301 (2012).

22. Markvoort, A. J., Eikelder, H. M. M. ten, Hilbers, P. A. J., Greef, T. F. A. de & Meijer, E. W. Theoretical models of nonlinear effects in two-component cooperative supramolecular copolymerizations. *Nat. Commun.* **2**, 509 (2011).

23. Mataga, N., Kaifu, Y. & Koizumi, M. The Solvent Effect on Fluorescence Spectrum, Change of Solute-Solvent Interaction during the Lifetime of Excited Solute Molecule. *BCSJ* **28**, 690–691 (1955).

24. Kawski, A. On the Estimation of Excited-State Dipole Moments from Solvatochromic Shifts of Absorption and Fluorescence Spectra. *Z. Naturforsch. A* **57**, 255–262 (2002).

25. Wang, C. *et al.* Twisted intramolecular charge transfer (TICT) and twists beyond TICT: from mechanisms to rational designs of bright and sensitive fluorophores. *Chem. Soc. Rev.* **50**, 12656–12678 (2021).

26. Sasaki, S., Drummen, G. P. C. & Konishi, G. Recent advances in twisted intramolecular charge transfer (TICT) fluorescence and related phenomena in materials chemistry. *J. Mater. Chem. C* **4**, 2731–2743 (2016).

27. Chen, C. & Fang, C. Fluorescence Modulation by Amines: Mechanistic Insights into Twisted Intramolecular Charge Transfer (TICT) and Beyond. *Chemosensors* **11**, 87 (2023).

28. Sutar, P. *et al.* Controlling Molecular Packing in Aqueous Metallosupramolecular Self-assembly by Ligand Geometry. *Precis. Chem.* **1**, 332–340 (2023).
